# Listen to Genes: Dealing with Microarray Data in the Frequency Domain

**DOI:** 10.1371/journal.pone.0005098

**Published:** 2009-04-06

**Authors:** Jianfeng Feng, Dongyun Yi, Ritesh Krishna, Shuixia Guo, Vicky Buchanan-Wollaston

**Affiliations:** 1 Centre for Computational System Biology, Shanghai, Fudan University, Shanghai, People's Republic of China; 2 Department of Computer Science, Warwick University, Coventry, United Kingdom; 3 Department of System Science and Mathematics, National University of Defence Technology, Changsha, People's Republic of China; 4 Department of Mathematics, Hunan Normal University, Changsha, People's Republic of China; 5 Warwick HRI, University of Warwick, Wellesbourne, United Kingdom; Fondazione Telethon, Italy

## Abstract

**Background:**

We present a novel and systematic approach to analyze temporal microarray data. The approach includes normalization, clustering and network analysis of genes.

**Methodology:**

Genes are normalized using an error model based uniform normalization method aimed at identifying and estimating the sources of variations. The model minimizes the correlation among error terms across replicates. The normalized gene expressions are then clustered in terms of their power spectrum density. The method of complex Granger causality is introduced to reveal interactions between sets of genes. Complex Granger causality along with partial Granger causality is applied in both time and frequency domains to selected as well as all the genes to reveal the interesting networks of interactions. The approach is successfully applied to Arabidopsis leaf microarray data generated from 31,000 genes observed over 22 time points over 22 days. Three circuits: a circadian gene circuit, an ethylene circuit and a new global circuit showing a hierarchical structure to determine the initiators of leaf senescence are analyzed in detail.

**Conclusions:**

We use a totally data-driven approach to form biological hypothesis. Clustering using the power-spectrum analysis helps us identify genes of potential interest. Their dynamics can be captured accurately in the time and frequency domain using the methods of complex and partial Granger causality. With the rise in availability of temporal microarray data, such methods can be useful tools in uncovering the hidden biological interactions. We show our method in a step by step manner with help of toy models as well as a real biological dataset. We also analyse three distinct gene circuits of potential interest to Arabidopsis researchers.

## Introduction

Uncovering the biological meaning embedded in time-series gene expression data is one of the most challenging problems in the post genomic era. In comparison with fixed single time point microarray data, the expression patterns observed over multiple time periods provide us with a rich set of information detailing the temporal profiles of the genes. Such profiles when studied at the genome wide level can help us fully understand the underlying cellular processes and facilitate the development of potential therapeutic targets. Temporal analysis of microarray data has not only helped in identification of functional categories of genes but also in understanding the behaviour of various gene circuits. Techniques like Fourier estimations have been used for detection of periodic signals in various organisms including yeast and human cells [1], [2], [3]. Claridge-Chang et al. [4] used Fourier components to determine a set of genes expressed with a robust circadian rhythm in adult Drosophila head. Similar microarray study on circadian rhythm in Arabidopsis was carried out by Harmen et al.[5] which empirically tested for statistically significant cross-correlation between temporal profile of each gene and cosine wave of definite period and phase. Temporal microarray data has also been helpful in understanding the gene circuits using methods like Ordinary Differential Equations [6] and Dynamic Bayesian networks [7], [8]. The present study is based on the temporal gene expression data available for *Arabidopsis thaliana*, but our approach is general and can be directly applied to tackle other temporal gene microarray data.

The first step in dealing with microarray data is to process the data using an appropriate normalization technique. The normalization can help us deal with unwanted systematic variations associated with each biological sample, dye effects, gene selection bias, experimental conditions, human errors etc. We apply a normalization method inspired by [9], [10] on our custom designed microarrays. We realized that there was a problem in direct application of the method on our microarray platforms as it induced negative correlation among data. This issue can be of serious statistical concern while processing the data further. To avoid this bias due to correlation, a further approach is introduced to achieve a better normalization result. We term this approach *uniform normalization*.

The next obvious step after normalization is clustering of data to reduce the data dimension. Three popular methods [11] for clustering microarray data are : a) Point-wise distance based methods [12] b) model-based clustering methods [13] and c) feature-based clustering methods [14], [15]. However to the best of our knowledge, all clustering methods reviewed above are *visual clustering* i.e. a gene is classified into one class according to its distance from the class centers. Here in this paper, we attempt to apply the idea of *auditory clustering* i.e. classifying a gene into one class according to its frequency profile. One may argue that this certainly belongs to feature based clustering method. However, since frequency is one of the most important features we use, it is clearly distinguishable from visual features, and we also know that there are huge advantages of dealing with temporal data in the frequency domain, we call our method *auditory clustering* approach.

After applying our frequency based clustering approach to the data, we use Partial Granger causality [16] to infer network structures for interactions among selected genes from within and across those clusters. Three gene circuits are analysed in this paper. The first circuit is the circadian circuit comprising of 7 genes (ELF4, TOC1, CCA1, LHY, PRR7, PRR9 and GI). The second circuit is the ethylene signalling circuit comprising of 16 genes, and the third circuit is a global gene profile circuit of 9 genes. For the circadian and ethylene circuit, our results are in agreement with experimental data. For all the circuits, we present the causality analysis in both the time and frequency domain. Corresponding to double, triple and quadruple LOF (loss of function) mutation results, we introduce complex Granger causality here. In both the circadian and ethylene circuit, we find that the complex Granger causality plays an important role in reconciling experimental and computational results. Interactions in the global circuit is one of the most interesting results. To answer questions like, if there is a global picture of interactions among genes, we first simply cluster the genes using the k-mean method. Then we use the cluster centers (means) as representatives of each cluster and apply the Partial Granger causality to infer the interaction pattern. We see a clear hierarchical structure of interactions among the genes. At the top of the hierarchy are the genes with a peak in the middle, at the middle level of the hierarchy there are genes with a decreasing trend, and at the bottom level the genes exhibit an increasing trend.

## Methods

### Experimental details

Plant growth and leaf material acquisition: Arabidopsis (COL-0) was grown in controlled environment at 20°C, 70% relative humidity, 250 µmol m^−2^ s^−1^ light intensity, 16 h day length. Leaf 7 was tagged on emergence and biological replicates were harvested both morning and evening (7 h and 14 h into light period) at 2 day intervals until fully senescent. This resulted in 22 time point samples from before full leaf expansion to senescence.

RNA isolation and probe preparation: RNA was isolated from 4 individual leaves as separate biological replicates using the Triazol method (Invitrogen) followed by RNeasy column purification (Qiagen). RNA was amplified using a MessageAmp II (Ambion) and then labelled with Cy3 or Cy5 using reverse transcriptase (SuperScript II, Invitrogen). Each amplified RNA sample was labelled twice with Cy3 and twice with Cy5 giving 4 technical replicates for each leaf sample. Two Cy3 and C5 labelled samples (in 25% formamide, 5× SSC, 0.1% SDS and 0.5 mg ml^−1^ yeast tRNA) were mixed in different combinations for hybridization to microarray slides.

Microarray analysis: Microarrays (CATMA) carrying 31,000 Arabidopsis gene probes (constructed in house as described in [17]) were hybridized with labeled samples at 42°C overnight. Slides were washed and then scanned using an Affymetrix 428 array scanner at 532 nm (Cy3) and 635 nm (Cy5). Scanned data were quantified using Imagene version 7 software (BioDiscovery, http://www.biodiscovery.com/). Individual text files quantifying the output for Cy3 and Cy5 were used in the further data analysis.

### Normalizing: uniform normalization

It is basically assumed that the number of up-regulated and down-regulated genes is about the same, which does not usually hold for customized arrays due to gene selection biases. With the increased popularity of customized microarrays, which enables us to focus only on hundreds of genes of their primary interest with more reliable measurements, a certain gene bias exists which requires more sophisticated normalization techniques. The validation tests are essential for controlling the quality of downstream statistical data analysis of customized arrays.

Our study is motivated by the aforementioned fundamental concerns. We first estimate the various sources of variations in an experiment and if microarray data have been properly normalized, there should be no systematic biases among estimated values of genes. Therefore, the residuals associated with genes in a replicate, standardized by the estimated gene wise variances, should show a normal distribution. Also, the correlation between residuals from one replicate to other replicate should be minimum. A detailed normalization approach is presented in Text S1. The main difference between our approach and the approach existing in the literature lies in the fact that the normalized data set is less negatively correlated (see Results section). In fact, the correlation matrix of normalized data is much more ﬂat or uniform than the one without adopting our approach, hence the name uniform normalization.

### Clustering: auditory clustering

**Figure 1:**
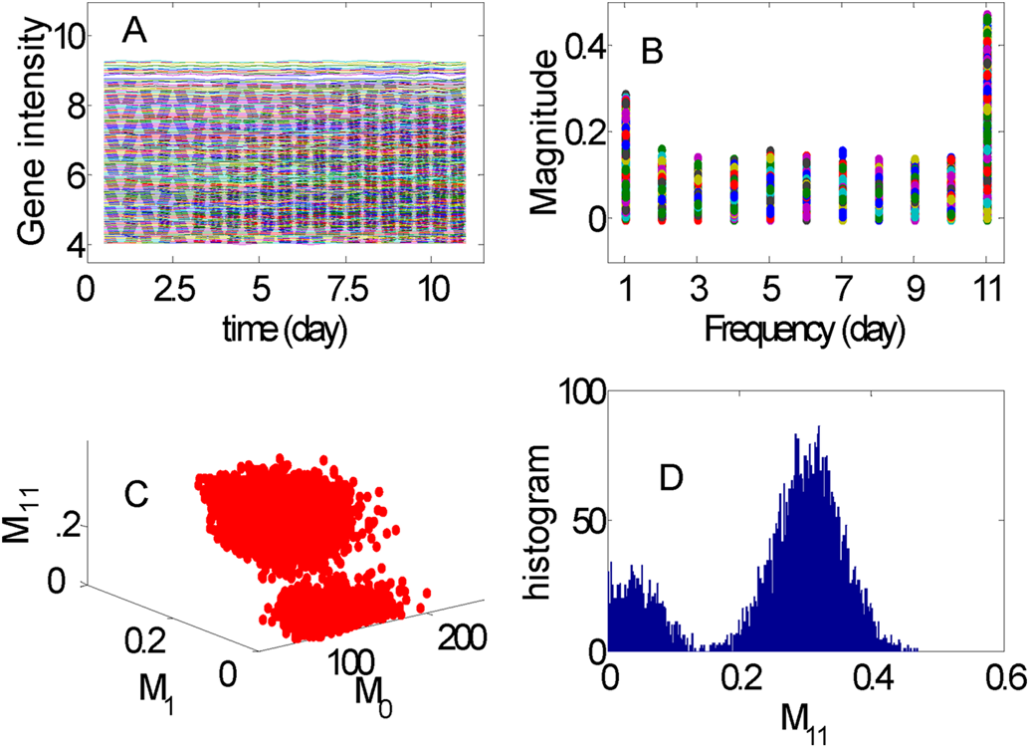
Synthesized data. A. Gene intensity vs. time. B. The magnitude of discrete Fourier transform of the data in A. The DC term is not shown. C. M0 (DC term), M1 (corresponding to the first column in B) and M11 (the 11th column in B). A clear structure of two clusters is shown. D. The histogram of the magnitude of M11.

The genes were clustered according to their frequency profiles. The clustering method is different from all existing clustering approaches for microarray data in the literature. As mentioned before, this method can be classified as *auditory clustering*. To fully illustrate the method, we use a toy model shown in Figure 1. In order to generate the simulation data, we randomly selected 3000 genes from the original dataset and computed their power spectrum. Major frequencies present in the system were for day 1 and 22. We used following equations to generate the simulation data having similar frequency components.
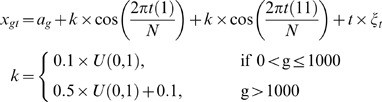



The term 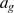 is the DC term computed for the gene g from original dataset after taking the Fourier transform. 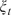 is the uniform error associated with simulated 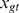.

The panel A in the Figure 1 plots the time domain representation of 3,000 genes. Though it is difficult to see the grouping of genes in the time domain representation, we transform the data to the frequency domain and the results are shown in Figure 1B. Two main, dominant frequencies corresponding to M1 and M11 can be seen in the Figure, M1 and M11 are the first and the 11^th^ components of the discrete Fourier transformation. Figure 1C also confirms that two different frequencies are present in the data, one in the high frequency (M11) and the other being in the low frequency (M1). The behaviour can also be seen in the Figure 1D. The functional meaning of the clusters is obvious. The genes with a higher M11 values are most sensitive to (controller of) faster changes, whereas the genes with higher M1 values are responsible for slower changes. In general, we can face a data set which has multiple frequencies. Each frequency has its own physiological meaning. For example in HeLa cell, we have reported that there are three dominant frequencies [16].

### Complex Granger causality: Network Analyzing

We have developed a novel approach called Partial Granger causality (PGC) to analyze network structures in genes, neurons and brain areas[16]. We adopted PGC approach here. However, when we examined the actual gene data, we found (see next section) that the data is definitely not stationary. In order to apply the PGC, we have to pre-process the data. The first, and also the simplest, one is to use ARIMA rather than ARMA model to fit the data and then apply the PGC to the ARIMA model. It is not difficult to see that the PGC in an ARMA model and PGC obtained from an ARIMA model is equivalent.

Interactions between two groups of genes (complex) are also introduced to emulate the multi-gene mutation experiments . The complex interactions are considerably different from interactions observed at pair wise level. For example, a pair of nodes may not have any individual interaction with the third node, but when in combination with each other, they may interact with the third one. On the other hand, when two nodes are negatively correlated, each of them can interact with the third one, but when they are grouped together, the interaction may disappear. We will direct the readers to look at the Text S5 for a detailed discussion on this topic.

For two vectors (groups of genes at time t) (X(t),Y (t)), Y (t) is a Granger cause of X(t) if 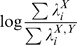
is significantly greater than zero, where 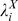 are eigenvalues of the residual matrix 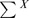


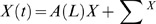
and 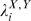
are eigenvalues of the residual matrix 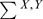


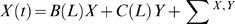
with L as the delay operator and A, B, C being appropriate polynomials. The 95% confidence intervals are constructed using the bootstrap method. An interaction between two genes or two group of genes is significant if and only if the low bound of the confidence interval is greater than zero.

## Results

### Normalization

**Figure 2:**
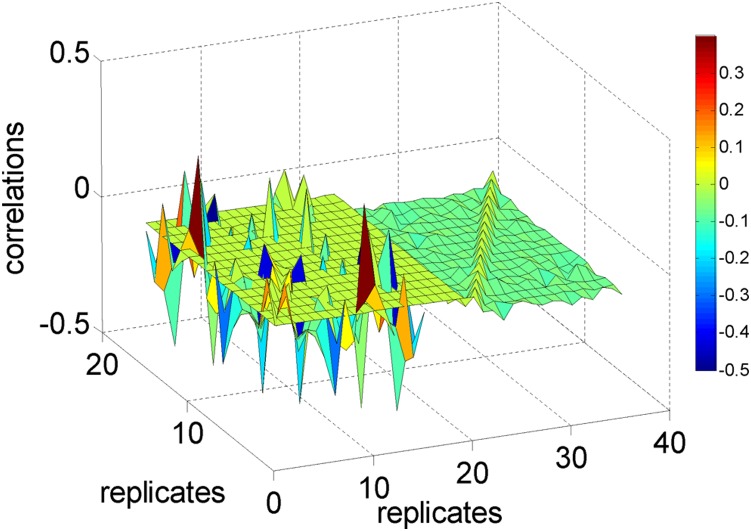
Correlation matrix before and after uniform normalization. For x = 1, 2, ··· , 16 is the correlation matrix before applying the uniform normalization (see Text S1). For x = 21, 22, ··· , 36 is the correlation matrix after applying the uniform normalization (see Text S1). The diagonal elements of two matrices are all set to 0.

The correlation matrix of 16 replicates of the normalized data as mentioned in Method section and Text S1 is shown in Figure 2. The result obtained after removing the different biases is shown in x ∈ [1], [16]×y ∈ [1], [16]. The existence of negative correlation among the replicates can be seen in Figure 2 (more downward spikes than upward). After applying our method to the data, the negative correlation is evenly distributed over all replicates x ∈ [21, 36]×y ∈ [1,16]. This considerably improves the outcome of the normalization. For a detailed description of the method we refer the reader to the Text S1.

### Frequency analysis

**Figure 3:**
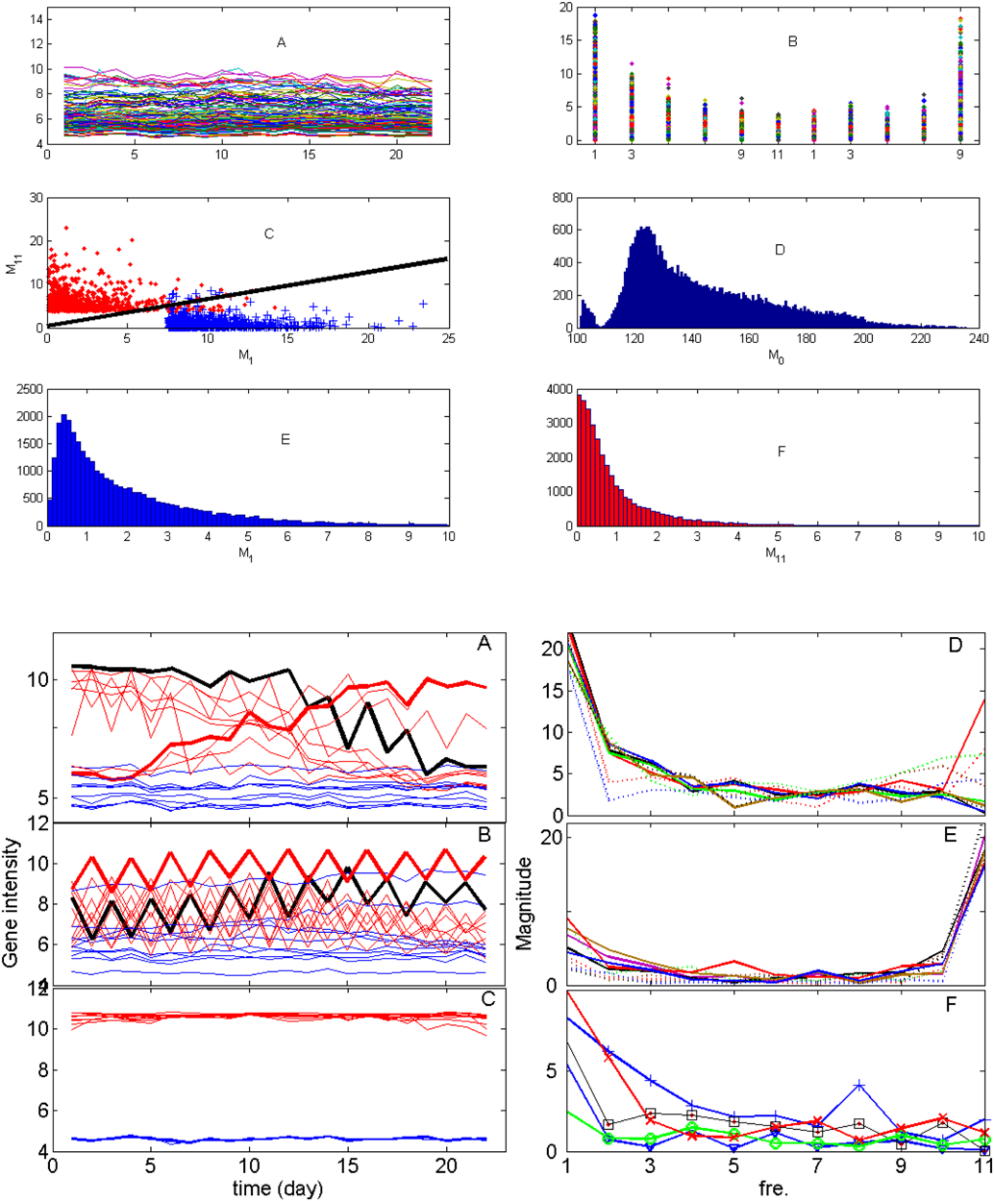
I Microarray data of 31,000 genes. IA. Gene intensity vs. time. Only 200 genes are shown. IB. Magnitude of all genes vs. frequency. It is clear to see that there are two main frequencies in the data, i.e. the one of one day period (M11, the 11th column) and the other of 22 days period (M1, the first column). The DC term M0 is not shown. IC. Two dimensional plot of M11 vs. M1. ID. The histogram of the DC term. There are two peaks in the histogram. IE. The histogram of M1, it is a Weibull distribution. IF. The histogram of M11, it is an exponential distribution. II. Time trace of top ten genes with 22-day, one day period and flat. IIA. Time trace of the first (in red and black) and bottom (in blue) ten genes with the strongest amplitude of the period of 22 days. There are two classes: one is up regulated (red thick line), the other is down regulated (black thick lines). IIB. Time trace of the first (in red and black) and bottom (in blue) ten genes with the strongest amplitude of period of 1 day. There are two classes: one is on-phase (red thick line), the other is off-phase (black thick line). IIC. Time trace of the first top (in red) and bottom (in blue) ten genes without rhythms. IID, IIE, IIF, the power corresponding to IIA, IIB and IIC respectively.

After normalizing the data (Figure 3IA), we turn our attention to create clusters in the frequency domain. It is important to note that a successful Fourier analysis depends on a careful design of experiment and data collection method. Too short data and or a collection on data points on irregular intervals can miss the natural cycles present in the system and the Fourier analysis may not be fruitful. The effect of windowing data for Fourier transformation is well understood in literature, see for example [18]. We took such important issues in consideration while collecting the data. First, our data is long enough and collected over 22 days which allows to capture lots of changes in gene expression profiles. Second, our data was collected to capture the cyclic behaviour due to daily activity (24 hour period) in the plant. Twice a day data collection also allowed us to monitor the gene expressions due to day and night effect. Though our data was not collected on smaller intervals which meant that we missed the smaller frequencies but the larger frequencies could still be captured and utilized for our purpose. We find two dominant frequencies in the data as shown in Figure 3IB; one with a period of one-day and the other with a period of 22-day (the power corresponding to x = 11 and the power corresponding to x = 1). In Figure 3IC, we plot M11 (all genes with M11>5) vs. M1 (all genes with M1>8). The thick line indicates that the genes with a strong one day rhythm are separate from the genes which have a rhythm of 22-day (see below in Figure 3II). Figure 3ID is the histogram of the DC term: it is interesting to see that it is a two-modal distribution. Most genes have a 22-day rhythm (Figure 3IE), in comparison with Figure 3IF.

In order to have a clear understanding of the power spectrum distribution, we plot top 10 and bottom 10 genes in Figure 3 IIA (according to M1), IIB (according to M11) and IIC (according to M0). Figure 3IIA plots the genes with a 22-day frequency showing the biggest jumps for 22 days. All the top 10 genes can be divided into two classes a) down regulated (6 genes) and b) up regulated (4 genes). One may infer that these genes could be closely related to senescence. The genes with maximum power of frequency at of one day are oscillatory genes (circadian genes). They can be further divided into two classes: in-phase (4 genes) and out-phase (6 genes) as shown in Figure 3IIB. Finally in Figure 3C, genes which are ﬂat are plotted. Figure 3IID, IIE and IIF are the corresponding power of Figure 3IIA, IIB and IIC.

### A Circadian Circuit

**Figure 4:**
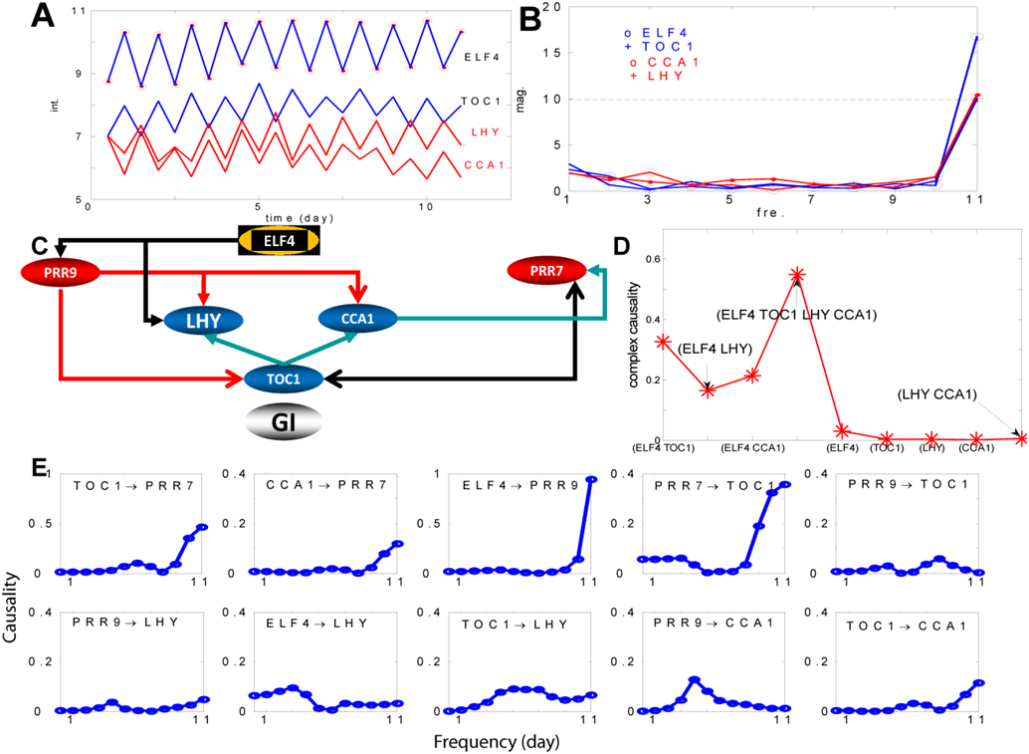
One gene circuit controlling circadian activity. A. Time trace of four genes, ELF4, TOC1, LFY and CCA1. ELF4 and TOC1 are in-phase oscillators, LFY and CCA1 are in-phase oscillators, but they are off-phase oscillators with respect to ELF4 and TOC1. B. Magnitudes vs. frequency for the four genes. They have highest magnitude at the frequency of one-day period. C. The gene circuit obtained in terms of PGC (see annotation in Text S2). D. Complex interactions between different group of genes and GI. E. Gene interactions in the frequency domain. The y-axis represents the strength of causal interactions.

In Figure 4A, the top most gene ELF4 shows a strong circadian rhythm. Actually it has the biggest M11 value. The importance of ELF4 in regulating the circadian activity is also reported in the literature [19], [20]. From the gene annotation (also presented in Text S2), we found that ELF4 is related to two other genes: LHY and CCA1. ELF4 is necessary for light-induced expression of both CCA1 and LHY. Figure 4A plots the time trace of these genes. A circadian circuit related to LHY and CCA1 has been reported in the literature [21], [22]. The circuit comprises of three loops; PRR9, PRR7 and LHY/CCA1 in one loop (morning loop or loop III), TOC1 and GI as another loop (night loop or loop II), and a loop of LHY/CCA1, TOC1 and an unknown gene as loop I.

We therefore consider a gene circuit of 7 genes (see Text S2). Four genes (ELF4, TOC1, LHY and CCA1) show a strong circadian rhythm as plotted in Figure 4B. We see that all genes have very strong magnitude on the period of 11 days. We applied partial Granger causality on those genes and the resulting network is shown in Figure 4C. The inferred structure is broadly in terms with the existing literature[23]. ELF4 plays an important role in regulating the circadian activity and is the most upstream genes. It interacts with both the loop III and the loop I. Loop III genes are closely interconnected via the interactions between PRR9, LHY and CCA1, and the interaction between CCA1 and PRR7. Similarly, in the loop I , TOC1 modulates LHY and CCA1. There are also links between loop III and loop I: PRR9 exerts influence on TOC1. TOC1 and PRR7 have a feedback loop. GI is an isolated gene in our structure, without having any interactions with other six genes. In fact, this also coincides with the experimental findings. On page 4 [23], it is mentioned that *The gi single mutant had a relatively weak phenotype*, *whereas our assays of the triple gi*; *lhy*;*cca1 mutant demonstrate GI*'*s importance*. This naturally leads us to introduce the notation of interactions between complexes as defined in the Method section. Fig. 4D tells us that all single genes ELF4, TOC1, LHY, CCA1 and (LHY, CCA1) have very little influence on GI. However, ELF4, TOC1, LHY and CCA1 together exhibit a significant interaction with GI. This is an example of how complex causality between sets of genes can be useful for deriving meaningful conclusions. A GO annotation table describing the discussed genes for circadian rhythm is presented in Table 1.

**Table 1:** GO annotations for the genes discussed for circadian rhythm.

Gene Number	Gene Name	GO Identifier	GO Term
At2g40080.1	ELF4 (EARLY FLOWERING 4)	GO:0042753	Positive regulation of circadian rhythm
At1g01060.1, At1g01060.2, At1g01060.3, At1g01060.4	LHY (LATE ELONGATED HYPOCOTYL)	GO:0048574	Long-day photoperiodism, flowering
At2g46830.1, At2g46830.2	CCA1 (CIRCADIAN CLOCK ASSOCIATED 1)	GO:0007623	Circadian rhythm
At5g61380.1	TOC1 (TIMING OF CAB1 1)	GO:0007623	Circadian rhythm
At5g02810.1	PRR7 (PSEUDO-RESPONSE REGULATOR 7)	GO:0007623	Circadian rhythm
At2g46790.1, At2g46790.2	PRR9 (PSEUDO-RESPONSE REGULATOR 9)	GO:0007623	Circadian rhythm
At1g22770.1	GI (GIGANTEA)	GO:0007623	Circadian rhythm

We then analyse the interactions in the frequency domain. Not surprisingly, almost all the interactions show a 24 hour periodic behaviour by exhibiting a peak at one day period.

### An Ethylene Signalling Pathway

**Figure 5:**
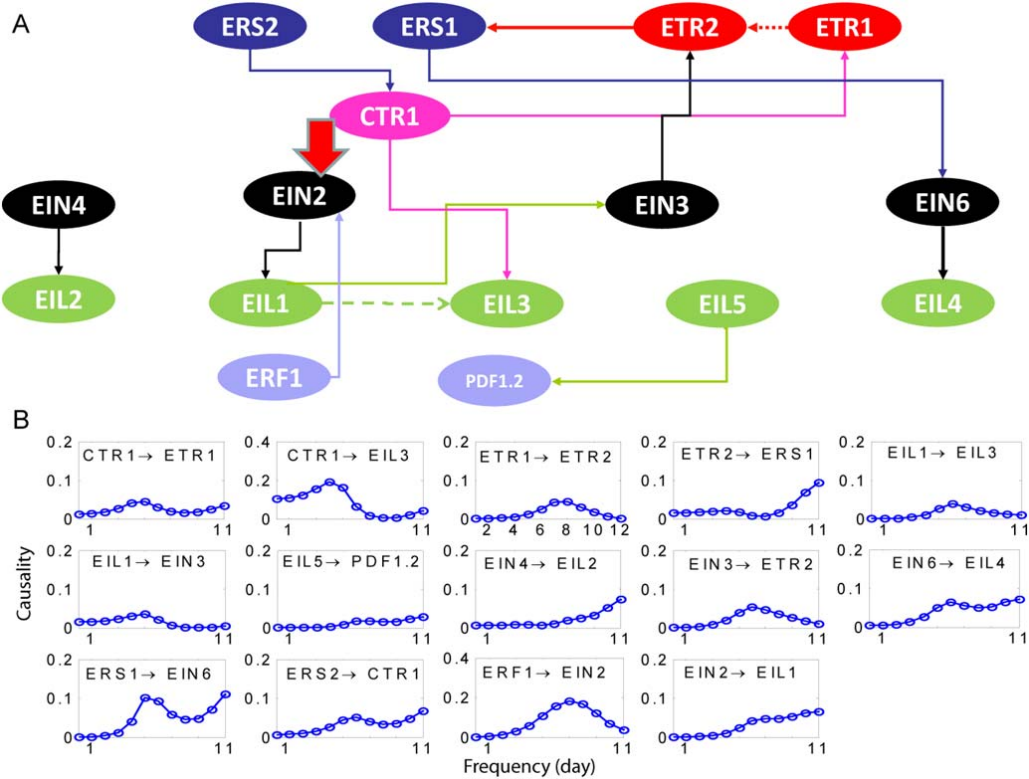
A circuit of ethylene pathway. A. An ethylene gene circuit with around 16 genes. Only genes with interactions are shown here. The thick arrow is the complex interaction between {CTR1, ETR1 and ERS2} and EIN2. B Interactions in the frequency domain calculated in terms of PGC. Only 14 significant interactions are shown.

Ethylene signalling pathway [24], [25] is one of the most well studied circuits in the literature due to its importance in myriad developmental processes and fitness responses. Here we selected a group of genes (16, see Text S3) which have been reported in the literature to play a central role in the pathway. Ethylene is perceived by a family of integral membrane receptors. In Arabidopsis, at least five family members are involved: ETR1, ETR2, ERS1, ERS2 and EIN4. ETR1 and ERS1 belong to type 1 receptors whereas EIN4, ETR2 and ERS2 are type 2 receptors. The receptors are hypothesized to be in a functionally active form that constitutively activates CTR1. It is reported that the interaction of type 1 receptors with CTR1 is stronger than type 2 receptors. CTR1 is an upstream gene, and has been reported as the regulator of the pathway [25]. In our inferred circuit, we obtain interactions of CTR1 with ERS2 and CTR1 with ETR1. Both are biologically verified [25]. Though EIN2 is an important component in the Ethylene circuit, its function is not completely understood [26]. It has been suggested that in the downstream of CTR1 and on the upstream of EIN2, a SIMKK-MPK6 pathway exists which may be regulated by CTR1, but this is yet to be verified biologically. So, we directly focus on the interactions between CTR1 and EIN2 and check whether CTR1 regulates EIN2 or not. To understand the interactions between CTR1 and EIN2, we chose to use complex causality by grouping together CTR1, ETR1 and ERS2; and analysing the interaction of the group with EIN2. We found that CTR1 does have a relationship with EIN2 and this is shown in the Figure 5A (thick arrow). EIN3 is most closely related to EIL1 [25] and this interaction can be found in the inferred network. Except two genes (EIN4 and EIL2) which are isolated and have no interactions with the rest of the genes, we see that the pathway shows a clear hierarchical structure. Interactions in the frequency are shown in 5B. Some interactions, for example ETR2 → ERS1, EIN6 → EIL4 etc., exhibit a strong daily rhythm.

### A Global Circuit

**Figure 6:**
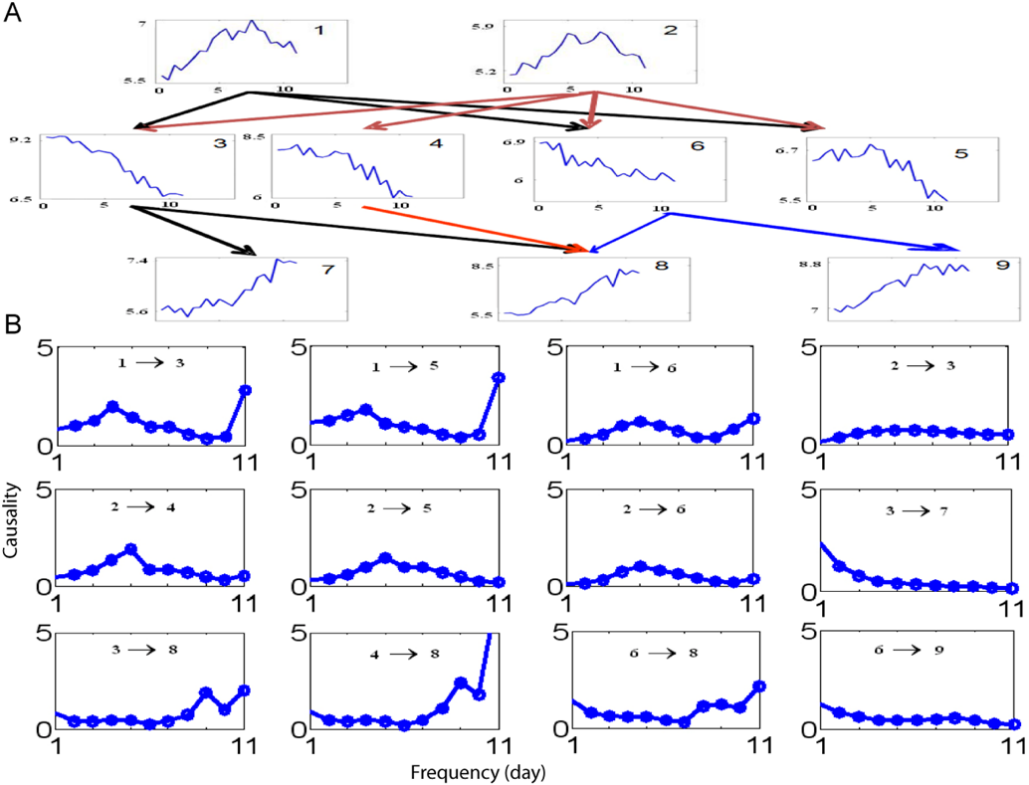
Causal relationship between genes: a global circuit. A. A total of 11 genes are shown and a clear hierarchy structure is demonstrated. B. The interactions in the frequency domain.

Finally we turn our attention to a global picture: to analyze the interaction network of all genes. In other words, to analyze how leaf senescence is turned on. All genes are clustered into clusters using the K-mean approach with a total number of different clusters (32, 20,··· etc). After clustering, we then pick up one gene or the centre to represent each cluster. The time trace of the representative gene is plotted in Fig. 6A (see Text S4 for examples of genes belonging to different clusters), together with their causality.

In Fig. 6A, it is clearly shown that the upstream genes exhibit a typical concave shape. All genes in the middle layer in the leaf senescence hierarchy have a peak at the beginning and then decrease. Finally the bottom layer genes increase their intensity during leaf senescence. The result fits our intuition very well. During the life time of a leaf, senescence associated genes are first expressed at a relative low intensity. Their intensity increases to their peak level, as an indication of the initiation of leaf senescence.

It is certainly surprising to see the stable global circuit underlying the all 31,000 genes. Its biological meaning is clear: leaf senescence is a stable process and is independent of a single or even a group of genes. Whether this is also true for other genomes (aging in mammals, for example) is a challenging and interesting issue. On the other hand, results in Fig. 6A also show us the power of our Granger causality approach. Intuitively, one would not expect that gene 1, for example in 6A, is the cause of gene 3 since the down-regulation of the gene 3 starts at an early time (day one).

In Fig. 6B, the interactions in the frequency domain are plotted. We can see that the interactions at the frequency domain are different between the top and middle layer and the middle layer and bottom layer. In general, we have a peak in the middle frequency between the top and the middle layer: see for example, 1 → 3. But the interactions between the middle and the bottom layer are concentrated on either the high or the low frequencies.

## Discussion

We have presented a complete work-flow for temporal microarray data processing. A fresh approach has been taken to accomplish each step in the work-flow ; from processing of raw data to gene network inference. This paper breaks new ground in normalization and clustering methods for highly replicated temporal microarray data. The normalization method allows each gene to be represented as identical and independent stochastic process, and the auditory clustering reduces the data dimension by applying simple but powerful frequency based approach. We have shown in the paper that the clustering method not only categorizes the genes according to their functionality but also allows a purely data driven natural ranking of genes based on their power spectrum profile. Two important concerns, namely, the optimal number of clusters in a dataset and ranking of each gene within each cluster are naturally handled using our method. We describe in the Frequency Analysis section that how the natural ranking of genes allowed us to select the genes involved in the circadian circuit of Arabidopsis. Encouraged by these results, we decided to study the circadian circuit in more detail and analyse it. We used our method of complex partial Granger causality to infer the gene interaction network for the circuit. Our time and frequency based analysis show that the computationally inferred network structure is in agreement with the experimental findings.

We further applied Partial Granger causality in time as well as frequency domain to selected genes involved in the ethylene pathway. In the end, we clustered the complete dataset of 31,000 genes with a standard k-mean clustering method to detect any pattern among the genes. After selecting a representative gene from each cluster, we applied Partial Granger causality to obtain a global interaction circuit. A clear hierarchical communication pattern emerged for the genes involved in the global circuit.

These are the first steps in applying a frequency domain approach to deal with temporal microarray data. There remain many issues to be further explored on the lines of frequency domain analysis. Is there any random gene (white signal) or a group of random genes having a flat PSD? The PSD is distributed according to Weilbull distribution. Is there an link between the life-span distribution of genes and PSD distribution? Here we only checked for the frequency domain interactions at an identical frequency. For a complex system, we expect that an interactions at different frequencies exists (see for example, [27]. In the frequency domain analysis, it is known that the most efficient way to nullify an input signal at a given frequency is by applying a filter. Can we develop biological *filter* to fulfil certain purposes, for example, to prolong the life span of a leaf? We have also microarray data of the Arabidopsis leaf respond to infection with the plant pathogen *Botrytis cinerea* available, with a time interval of 2 hours. A direct application of our approach seems very encouraging.

## Supporting Information

Text S1 Uniform Normalization
(0.10 MB PDF)

Text S2 Gene names and descriptions in circadian circuit.
(0.05 MB PDF)

Text S3 Gene names and descriptions in ethylene circuit.
(0.06 MB PDF)

Text S4 Selected gene names and descriptions for the global circuit.
(0.06 MB PDF)

Text S5 Complex Interactions
(0.45 MB PDF)
