# Supplementary material for: Listen to Genes: Dealing with Microarray Data in the Frequency Domain
Source: PLoS One. 2009 Apr 6;4(4):e5098. doi: 10.1371/journal.pone.0005098 (PMC3383793; doi:10.1371/journal.pone.0005098)
Supplement: Text S1 — Uniform Normalization (0.10 MB PDF) [file pone.0005098.s001.pdf]

Text S1: Uniform Normalization

Normalization

Microarray technologies provide a powerful mechanism to simultaneously detect and measure expression levels for tens of thoudands of genes in a single experiment. The vast quantity of data if suitably analyzed can help in understanding cellular processes, diagnosis of diseases and development of potential therapeutic targets. The effective analysis of the experimental results rely on the good quality of data. Experimental variations such as design of arrays, mRNA quality,labellings and dye effects, hybridization conditions, human and machine errors in scanning process contribute toward obscuring variations found in a Microarray data. To overcome these obscure variations, and make the observations from different array comparable, an effective normalization technique is required. A number of normalization methods have been proposed over the years but it is still an active field for research. While studying different normalization techniques one often comes across the assumption that various sources of biases in a microarray experiment are either completely confounded by each other or are orthogonal to each other.Confounded effects mean, one effect is estimable only when the other effect is zeros, whereas orthogonality of effects results in a scenario where including or excluding one effect in the model does not alter the estimates obtained for other effects. Global normalization methods also assume that the various biases are same across the experimental setup. To accommodate these idea in the mathematical framework, different biases are constrained to have zero mean value across microarray species population and replicates. While these constraints are important to obtain estimates and reason well with a carefully designed experiment, they are global in nature. If we concentrate on a local population in a experiment, these constraints may not hold together, and normalizing a local population can result in several concerns. One of the concerns being the presence of strong correlation among various species within a replicate and across the replicates, and the non-normal behaviour of residual terms. In this paper, we present a method to normalize global as well as local population of data by handling the correlation between species and replicates while controlling the variances and also correcting the residuals for normal behaviour.

Let Y_{gbi} be the log of observed gene expression for gene g, on biological sample b, measured on the replicate i. The reason to analyse the data value based on log scale is due to the fact that log transform is the natural method for analysing data with an additive model where the effects in the data are believed to be multiplicative. the common use of ratios to analyse microarray data also illustrates this assumption. To account for multiple sources of variations in a microarray experiment, consider the following model

$$Y_{gbi} = \alpha_g + \beta_b + \gamma_{bi} + M_{gbi} + \zeta_{gbi} \tag{1}$$

where α_g is the assumed true value of the gene expression, β_b is the systematic variation associated with each biological sample,γ_{bi} is the systematic variation associated with the replicate i for the biological sample b. M_{gbi} is the gene-specific effects of dyes, selection bias and other experimental conditions. Finally, ζ_{gbi} is the error term in the model. Our goal is to a) estimate all the above factors of variations, b) estimate the error term ζ_{gbi} such that it is independent and identically distributed with mean zero and constant variance and c) the error terms are least correlated across replicates. Here we assume that each gene is spotted only once on each array and the replicates include both biological and experimental replicates.

For simplicity, the model in Equation 1 can be expressed as

$$Y_{gbi} = \alpha_g + \beta_b + \gamma_{bi} + \epsilon_{gbi} \tag{2}$$

where

$$\epsilon_{gbi} = M_{gbi} + \zeta_{gbi} \tag{3}$$

For the further derivation, we fix $g = 1, 2, \ldots, G$, $b = 1, 2, \ldots, B$ and $i = 1, 2, \ldots, I$ associated with each b. We first present the following steps for estimation of γ_{bi}, β_b and α_g, and later we show how to process ϵ_{gbi} to achieve desired goals.

With each biological sample b and the replicate i associated with it, the average gene

expression for a gene g can be obtained as

$$\overline{Y}_{gb} = \frac{1}{I}\sum_{i=1}^{I} Y_{gbi} \tag{4}$$

The bias associated with each replicate i for a given b can be obtained by removing the effect of average gene expression $\overline{Y}_{gb}$ from each Y_{gbi}. Thus

$$Y_{gbi} - \overline{Y}_{gb} = \alpha_g + \beta_b + \gamma_{bi} - \alpha_g - \beta_b - \frac{1}{I}\sum_{i=1}^{I}\gamma_{bi} + \epsilon'_{gbi} \tag{5}$$

Using the Least-square estimates, the systematic variation γ_{bi} can be estimated as

$$\Rightarrow \widehat{\gamma_{bi}} = \frac{1}{G}\sum_{g=1}^{G}(Y_{gbi} - \overline{Y}_{gb}) \tag{6}$$

To estimate the variation β_b among biological samples we first remove the variation $\widehat{\gamma_{bi}}$ from the log of observed gene expression. Let

$$Y'_{gbi} = Y_{gbi} - \widehat{\gamma_{bi}}$$

hence according to our model in Equation 1

$$Y'_{gbi} = \alpha_g + \beta_b + \epsilon_{gbi}$$

Now for each b we have

$$Y''_{gbi} = Y'_{gbi} - \overline{Y}_{gi}$$

where $\overline{Y}_{gi}$ is the average gene expression for a given replicate i across all the biological samples $b = 1, 2, \ldots, B$.

$$Y''_{gbi} = \alpha_g + \beta_b - [\alpha_g + \frac{1}{B}\sum_{b=1}^{B}\beta_b] + \epsilon''_{gbi}$$

The values for $\hat{\beta}_b$ for $b = 1, 2, \ldots, B$ can be estimated using Least-squares and by averaging over all the genes $g = 1, 2, \ldots, G$. In the next step, we can remove the biological

variation β_b and the combined effect of variation of biological sample b and replicate i captured in $\widehat{\gamma_{bi}}$ from the Y_{gbi} to estimate the expected value of the gene expression α_g.

$$Y'''_{gbi} = Y_{gbi} - \alpha_g - \hat{\beta_b} - \hat{\gamma_{bi}} = \alpha_g + \epsilon_{gbi}$$

$$\hat{\alpha_g} = \frac{1}{B \times I} \sum_{b,i} Y'''_{gbi} \tag{7}$$

After estimating $\hat{\alpha_g}, \hat{\beta_b}$ and $\hat{\gamma_{bi}}$, we can compute $\hat{\epsilon_{gbi}}$ from our model in Equation 2 as

$$\hat{\epsilon_{gbi}} = Y_{gbi} - \hat{\alpha_g} - \hat{\beta_b} - \hat{\gamma_{bi}} \tag{8}$$

In our model β_b and γ_{bi} are variations specific to biological samples and replicates. But there may be many other sources of variations in an experiment which may be confounded in various combinations and are captured in the expression ϵ_{gbi} which is specific to each gene, biological sample and replicate. In the ideal conditions, ϵ_{gbi} should be independent and identically distributed and should be uncorrelated across replicates. But this seldom is the case because of presence of many other unknown sources of experimental variations in a dataset. Thus ϵ_{gbi} demands a separate analysis and treatment.

Recall that according to Equation 3, ϵ_{gbi} is composed of M_{gbi} and ζ_{gbi} which can be calculated separately. Consider a $G \times R$ matrix E having values of ϵ_{gbi}. G is the total number of genes and R are the total replicates present in the experiment. Let X be the $I \times I$ correlation matrix of E. In order to remove high degree of correlation among the values in E across different columns (corresponding to different replicates), we can apply an iterative procedure where the ϵ_{gbi} values for each replicate i denoted as the E_i column can be represented as a linear combination of highly correlated columns selected from the rest of the columns in E. The least correlated column can be decided by looking at the entries in the correlation matrix X. So in the beginning of the iteration,we denote

$$E_i = \Sigma S_k E_k + \zeta_i$$

where $k \neq i, j$ We compute the S_k coefficient and ζ_i with Least-square method and compute a correlation matrix of E_i with the E_k columns. We terminate the iteration if we

find all the correlation coefficients of E_i with the E_k columns to be less than 0.1, or if the variance of ζ_i has stabilised, or all the columns but one is remaining from the set of E_k columns. Otherwise, we drop a least correlated column from E_k columns and iterate the process till the termination criteria is met. In the end, we store $\Sigma S_k E_k$ as M_{gbi} and ζ_i as ζ_{gbi} to be introduced in the Equation 1.

Upon inspection of the results, we found that though the correlation among replicates drop significantly, but there is presence of more negatively correlated ζ_{gbi} terms compared to positively correlated ones. In order to deal with the skewness in the correlation terms, we distribute the correlation in system equally among all the replicates. Assuming ζ_{gbi} as

$$\zeta_{gbi} = \zeta_{gbi} - a_l\zeta_{gbi} + a_l\zeta_{gbi}$$

and further

$$\theta_{gbi} = \zeta_{gbi} - a_l\zeta_{gbi}$$

So

$$\zeta_{gbi} = \theta_{gbi} + a_l\zeta_{gbi} \tag{9}$$

θ_{gbi} is the white noise element. By denoting the matrices having θ_{gbi} elements as θ, a_i elements as A and ζ_{gbi} matrix as ζ we have,

$$\zeta = \theta + A\zeta \tag{10}$$

The correlation matrix (ρ) of θ across all the replicates R has approximately the same correlation coefficient

$$\rho = \begin{bmatrix} 1 & \frac{-1}{R-1} & \dots \\ \frac{-1}{R-1} & 1 & \dots \\ \vdots & \vdots \ddots & \end{bmatrix}$$

The unknown matrix A can be computed from Equation 10 where

$$\theta = (I - A)\zeta$$

Denoting the covariance matrix of θ and ζ as Σ_θ and Σ_ζ respectively, we have

$$A = I - \sqrt{(}\Sigma_\theta)(\sqrt{(}\Sigma_\zeta))^{-1} \tag{11}$$

So eventually,the final model in terms of Equation 1, after removing all the effects of variations and further breaking the error terms ζ_{gbj} in a way to equally distribute the remaining correlation in the system, can be expressed as

$$Y_{gbi} = \alpha_g + \theta_{gbi} \tag{12}$$

Correlation coefficients of residuals

1	0.16	-0.06	-0.03	-0.06	-0.14	-0.29	0.06	-0.11	0.41	0.10	-0.00	-0.10	0.12	-0.19	-0.03
0.16	1	0.07	-0.09	-0.06	-0.07	-0.00	-0.13	-0.16	-0.03	0.014	-0.03	-0.07	-0.11	-0.23	-0.15
-0.06	0.07	1	0.00	-0.12	-0.09	0.02	0.06	-0.07	0.00	-0.09	0.05	-0.01	-0.11	-0.44	0.00
-0.03	-0.09	0.00	1	0.06	-0.20	-0.01	-0.09	-0.00	-0.02	-0.09	-0.20	0.22	0.07	0.21	-0.03
-0.06	-0.06	-0.12	0.06	1	0.08	-0.42	-0.08	-0.05	-0.16	0.00	-0.069	-0.04	-0.07	-0.21	-0.11
-0.14	-0.07	-0.09	-0.20	0.08	1	0.04	0.10	-0.14	-0.18	-0.05	-0.13	-0.14	-0.50	0.00	-0.18
-0.29	-0.00	0.02	-0.01	-0.42	0.04	1	0.02	-0.12	-0.14	0.05	-0.19	0.03	-0.11	0.11	0.03
0.06	-0.13	0.06	-0.09	-0.08	0.10	0.02	1	-0.24	-0.10	-0.12	-0.09	-0.20	-0.00	-0.09	0.00
-0.11	-0.16	-0.07	-0.00	-0.05	-0.14	-0.12	-0.24	1	-0.06	-0.00	-0.09	0.07	-0.12	0.00	0.00
0.41	-0.03	0.00	-0.02	-0.16	-0.18	-0.14	-0.10	-0.06	1	-0.16	0.22	-0.09	0.01	-0.18	-0.09
0.10	0.01	-0.09	-0.09	0.00	-0.05	0.05	-0.12	-0.00	-0.16	1	-0.03	0.09	-0.09	-0.04	-0.08
-0.00	-0.03	0.05	-0.20	-0.06	-0.13	-0.19	-0.09	-0.09	0.22	-0.03	1	0.01	-0.08	-0.23	-0.13
-0.10	-0.07	-0.01	0.22	-0.04	-0.14	0.03	-0.20	0.07	-0.09	0.09	0.01	1	-0.08	-0.06	-0.08
0.12	-0.11	-0.11	0.07	-0.07	-0.50	-0.11	-0.00	-0.12	0.01	-0.09	-0.08	-0.08	1	0.00	0.04
-0.19	-0.23	-0.44	0.21	-0.21	0.00	0.11	-0.09	0.00	-0.18	-0.04	-0.23	-0.06	0.00	1	0.12
-0.03	-0.15	0.00	-0.03	-0.11	-0.18	0.03	0.00	0.00	-0.09	-0.08	-0.13	-0.08	0.04	0.12	1

Table 1: Correlation coefficients across replicates for ζ_{gbi}

1	-0.062	-0.063	-0.106	-0.049	-0.045	-0.063	-0.070	-0.059	-0.068	-0.088	-0.062	-0.077	-0.040	-0.049	-0.077
-0.062	1	-0.067	-0.060	-0.070	-0.072	-0.067	-0.066	-0.068	-0.066	-0.062	-0.067	-0.064	-0.073	-0.070	-0.064
-0.063	-0.067	1	-0.063	-0.069	-0.070	-0.067	-0.066	-0.067	-0.066	-0.064	-0.067	-0.065	-0.071	-0.069	-0.065
-0.106	-0.060	-0.063	1	-0.042	-0.036	-0.063	-0.072	-0.057	-0.069	-0.097	-0.061	-0.082	-0.030	-0.043	-0.082
-0.049	-0.070	-0.069	-0.042	1	-0.083	-0.069	-0.065	-0.072	-0.066	-0.053	-0.070	-0.060	-0.087	-0.079	-0.060
-0.045	-0.072	-0.070	-0.036	-0.083	1	-0.070	-0.064	-0.074	-0.066	-0.050	-0.071	-0.059	-0.092	-0.083	-0.059
-0.063	-0.067	-0.067	-0.063	-0.069	-0.070	1	-0.066	-0.067	-0.066	-0.064	-0.067	-0.065	-0.071	-0.069	-0.065
-0.070	-0.066	-0.066	-0.072	-0.065	-0.064	-0.066	1	-0.065	-0.066	-0.069	-0.066	-0.067	-0.064	-0.065	-0.067
-0.059	-0.068	-0.067	-0.057	-0.072	-0.074	-0.067	-0.065	1	-0.066	-0.061	-0.068	-0.063	-0.076	-0.072	-0.063
-0.068	-0.066	-0.066	-0.069	-0.066	-0.066	-0.066	-0.066	-0.066	1	-0.067	-0.066	-0.067	-0.066	-0.066	-0.067
-0.088	-0.062	-0.064	-0.097	-0.053	-0.050	-0.064	-0.069	-0.061	-0.067	1	-0.063	-0.074	-0.046	-0.053	-0.074
-0.062	-0.067	-0.067	-0.061	-0.070	-0.071	-0.067	-0.066	-0.068	-0.066	-0.063	1	-0.064	-0.072	-0.070	-0.064
-0.077	-0.064	-0.065	-0.082	-0.060	-0.059	-0.065	-0.067	-0.063	-0.067	-0.074	-0.064	1	-0.057	-0.060	-0.070
-0.040	-0.073	-0.071	-0.030	-0.087	-0.092	-0.071	-0.064	-0.076	-0.066	-0.046	-0.072	-0.057	1	-0.086	-0.057
-0.049	-0.070	-0.069	-0.043	-0.079	-0.083	-0.069	-0.065	-0.072	-0.066	-0.053	-0.070	-0.060	-0.086	1	-0.060
-0.077	-0.064	-0.065	-0.082	-0.060	-0.059	-0.065	-0.067	-0.063	-0.067	-0.074	-0.064	-0.070	-0.057	-0.060	1

Table 2: Correlation coefficients across replicates for θ_{gbi}
