# Supplementary material for: Listen to Genes: Dealing with Microarray Data in the Frequency Domain
Source: PLoS One. 2009 Apr 6;4(4):e5098. doi: 10.1371/journal.pone.0005098 (PMC3383793; doi:10.1371/journal.pone.0005098)
Supplement: Text S2 — Gene names and descriptions in circadian circuit. (0.05 MB PDF) [file pone.0005098.s002.pdf]

Text S2: Gene Annotations

Gene Number	Gene Name	Description
At2g40080.1	ELF4 (EARLY FLOWERING 4)	Encodes a novel nuclear 111 amino-acid phytochrome-regulated component of a negative feedback loop involving the circadian clock central oscillator components CCA1 and LHY. ELF4 is necessary for light-induced expression of both CCA1 and LHY, and conversely, CCA1 and LHY act negatively on light-induced ELF4 expression. ELF4 promotes clock accuracy and is required for sustained rhythms in the absence of daily light/dark cycles. It is involved in the phyB-mediated constant red light induced seedling de-etiolation process and may function to coregulate the expression of a subset of phyB-regulated genes.
At1g01060.1, At1g01060.2, At1g01060.3, At1g01060.4	LHY (LATE ELONGATED HYPOCOTYL)	myb-related putative transcription factor involved in circadian rhythm along with another myb transcription factor CCA1
At2g46830.1, At2g46830.2	CCA1 (CIRCADIAN CLOCK ASSOCIATED 1)	Transcription factor; encodes a transcriptional repressor that performs overlapping functions with LHY in a regulatory feedback loop that is closely associated with the circadian oscillator of ARABIDOPSIS.
At5g61380.1	TOC1 (TIMING OF CAB1 1)	Transcription regulator; pseudo response regulator involved in the generation of circadian rhythms. TOC1 appears to shorten the period of circumnutation speed. TOC1 contributes to the plant fitness (carbon fixation, biomass) by influencing the circadian clock period.
At5g02810.1	PRR7 (PSEUDO-RESPONSE REGULATOR 7)	Transcription regulator; PRR7 and PRR9 are partially redundant essential components of a temperature-sensitive circadian system. CCA1 and LHY had a positive effect on PRR7 expression levels.
At2g46790.1, At2g46790.2	PRR9 (PSEUDO-RESPONSE REGULATOR 9)	Pseudo-response regulator PRR9. Involved in clock function. PRR7 and PRR9 are partially redundant essential components of a temperature-sensitive circadian system. CCA1 and LHY had a positive effect on PRR9. Interact with TOC1 in a yeast two-hybrid assay.
At1g22770.1	GI (GIGANTEA)	Together with CONSTANTS (CO) and FLOWERING LOCUS T (FT), GIGANTEA promotes flowering under long days in a circadian clock-controlled flowering pathway. GI acts earlier than CO and FT in the pathway by increasing CO and FT mRNA abundance. Located in the nucleus. Regulates several developmental processes, including photoperiod-mediated flowering, phytochrome B signaling, circadian clock, carbohydrate metabolism, and cold stress response. The gene's transcription is controlled by the circadian clock and it is post-transcriptionally regulated by light and dark.

Table 3: Gene names and descriptions in circadian circuit.
