# Supplementary material for: Listen to Genes: Dealing with Microarray Data in the Frequency Domain
Source: PLoS One. 2009 Apr 6;4(4):e5098. doi: 10.1371/journal.pone.0005098 (PMC3383793; doi:10.1371/journal.pone.0005098)
Supplement: Text S3 — Gene names and descriptions in ethylene circuit. (0.06 MB PDF) [file pone.0005098.s003.pdf]

# Text S3: Gene Annotations

| Gene Number | Gene Name | Description |
|---|---|---|
| At5g03730.1 ,At5g03730.2 | CTR1 (CONSTITUTIVE TRIPLE RESPONSE 1) | Kinase; Homologous to the RAF family of serine/threonine protein kinases. Negative regulator in the ethylene signal transduction pathway. Interacts with the putative ethylene receptors ETR1 and ERS. Constitutively expressed. |
| At1g66340.1 | ETR1 (ETHYLENE RESPONSE 1) | Two-component response regulator; Similar to prokaryote sensory transduction proteins. Contains a histidine kinase and a response regulator domain. Homodimer. Membrane component. Binds ethylene. Mutations affect ethylene binding and metabolism of other plant hormones such as auxin, cytokinins, ABA and gibberellic acid. Ethylene receptor. Has histidine kinase activity. |
| At4g20880.1 | ERT2 | Ethylene-responsive nuclear protein / ethylene-regulated nuclear protein; similar to ethylene-responsive nuclear protein - related [Arabidopsis thaliana] (TAIR:AT5G44350.1); similar to IMP dehydrogenase/GMP reductase [Medicago truncatula] (GB:ABE90052.1) |
| At3g23240.1 | ERF1 (ETHYLENE RESPONSE FACTOR 1) | DNA binding / transcription factor/ transcriptional activator; encodes a member of the ERF (ethylene response factor) subfamily B-3 of ERF/AP2 transcription factor family (ERF1). The protein contains one AP2 domain. There are 18 members in this subfamily including ATERF-1, ATERF-2, AND ATERF-5. EREBP like protein that binds GCC box of ethylene regulated promoters such as basic chitinases. Constitutive expression of ERF1 phenocopies ethylene over production. Involved in ethylene signaling cascade,downstream of EIN2 and EIN3. |
| At3g04580.1 ,At3g04580.2 | EIN4 (ETHYLENE INSENSITIVE 4) | Ethylene receptor, subfamily 2. Has serine kinase activity. |
| At3g20770.1 | EIN3 (ETHYLENE INSENSITIVE3) | Transcription factor; ethylene-insensitive3 |
| At5g03280.1 | EIN2 (ETHYLENE INSENSITIVE 2) | Transporter; Involved in ethylene signal transduction. Acts downstream of CTR1. |
| At5g21120.1 | EIL2 (ETHYLENE INSENSITIVE3-LIKE 2) | Transcription factor; ethylene-insensitive3-like2 (EIL2) |

*Continued on next page*

| | | |
|---|---|---|
| At2g27050.1 | EIL1 (ETHYLENE INSENSITIVE3-LIKE 1) | Transcription factor; ethylene-insensitive3-like1 (EIL1) |
| At1g73730.1 | EIL3 (ETHYLENE INSENSITIVE3-LIKE3) | Transcription factor; Encodes a putative transcription factor involved in ethylene signalling. Isolated DNA binding domain has been shown to bind DNA in vitro. |
| At5g10120.1 | Identical to Putative ETHYLENE-INSENSITIVE3-like 4 protein (EIL4) | Similar to ethylene insensitive 3 family protein [Arabidopsis thaliana] (TAIR:AT5G65100.1); similar to 52O08_27 [Brassica rapa subsp. pekinensis] (GB:AAZ67573.1); contains InterPro domain Ethylene insensitive 3; (InterPro:IPR006957) |
| At1g04310.1 | ERS2 (ETHYLENE RESPONSE SENSOR 2) | receptor; encodes an ethylene receptor related to bacterial two-component histidine kinases. |
| At2g40940.1 | ERS1 (ETHYLENE RESPONSE SENSOR 1) | receptor; Ethylene receptor, subfamily 1. Has histidine kinase activity. |
| At3g33520.1 | EIN6 | structural constituent of cytoskeleton; Encodes ACTIN-RELATED PROTEIN6 (ARP6), a putative component of a chromatin-remodeling complex. Required for both histone acetylation and methylation of the FLC chromatin in Arabidopsis. Located at specific regions of the nuclear periphery. Expression throughout plants shown by in-situ and immunolocalization methods. Mutants show defects in fertility, leaf, flower and inflorescence development and shorter flowering times. |
| At1g55010.1 | PDF1.2 | similar to PDF 1.5 ; similar to Cysteine-rich antifungal protein 4 precursor (AFP4) (GB:O24331); contains InterPro domain Gamma thionin; (InterPro:IPR008176) |
| At5g65100.1 | ethylene insensitive 3 family protein; Identical to Putative ETHYLENE-INSENSITIVE3-like 5 protein (EIL5) | similar to ethylene insensitive 3 family protein [Arabidopsis thaliana] (TAIR:AT5G10120.1); similar to 52O08_27 [Brassica rapa subsp. pekinensis] (GB:AAZ67573.1); contains InterPro domain Ethylene insensitive 3; (InterPro:IPR006957) |

*Continued on next page*

Table 4: Gene names and descriptions in ethylene circuit.
