# Supplementary material for: Listen to Genes: Dealing with Microarray Data in the Frequency Domain
Source: PLoS One. 2009 Apr 6;4(4):e5098. doi: 10.1371/journal.pone.0005098 (PMC3383793; doi:10.1371/journal.pone.0005098)
Supplement: Text S4 — Selected gene names and descriptions for the global circuit. (0.06 MB PDF) [file pone.0005098.s004.pdf]

Text S4: Gene Annotations

Gene Name	Description
At2g34960.1	CAT5 (CATIONIC AMINO ACID TRANSPORTER 5);cationic amino acid transporter; Arabidopsis thaliana amino acid permease family protein (At2g34960)
At3g09900.1 AtRABE1e / AtRab8E	Arabidopsis Rab GTPase homolog E1e; GTP binding; similar to AtRABE1d/AtRab8C, GTP binding [Arabidopsis thaliana] (TAIR:AT5G03520.1); similar to ras-related protein RAB8-3 [Nicotiana tabacum] (GB:BAB84324.1); contains InterPro domain Small GTP-binding protein domain; (InterPro:IPR005225); contains InterPro domain Ras small GTPase, Rab type; (InterPro:IPR003579); contains InterPro domain Sigma-54 factor, interaction region; (InterPro:IPR002078); contains InterPro domain Ras GTPase; (InterPro:IPR001806); contains InterPro domain Ras; (InterPro:IPR013753)
At4g38495.1	Unknown protein; similar to conserved hypothetical protein [Aedes aegypti] (GB:EAT47050.1); similar to OSIGBa0138E08-OSIGBa0161L23.9 [Oryza sativa (indica cultivar-group)] (GB:CAH67928.1); similar to Os04g0274400 [Oryza sativa (japonica cultivar-group)] (GB:NP_001052351.1); contains InterPro domain YL1 nuclear, C-terminal; (InterPro:IPR013272)
At1g03550.1	Secretory carrier membrane protein (SCAMP) family protein; similar to secretory carrier membrane protein (SCAMP) family protein [Arabidopsis thaliana] (TAIR:AT2G20840.1); similar to similarity to SCAMP37 [Pisum sativum] (GB:AAC82326.1); similar to Os01g0780500 [Oryza sativa (japonica cultivar-group)] (GB:NP_001044437.1); similar to Os07g0564600 [Oryza sativa (japonica cultivar-group)] (GB:NP_001060004.1); contains InterPro domain SCAMP; (InterPro:IPR007273)
At5g21950.1	Hydrolase, alpha/beta fold family protein; similar to hydrolase, alpha/beta fold family protein [Arabidopsis thaliana] (TAIR:AT4G33180.1); similar to Alpha/beta hydrolase fold [Medicago truncatula] (GB:ABE81749.1); contains InterPro domain Esterase/lipase/thioesterase; (InterPro:IPR000379); contains InterPro domain Alpha/beta hydrolase fold-1; (InterPro:IPR000073); contains InterPro domain Alpha/beta hydrolase; (InterPro:IPR003089)
At1g53170.1	ATERF-8/ATERF8 (ETHYLENE RESPONSE ELEMENT BINDING FACTOR 4); DNA binding / transcription factor/ transcriptional repressor; encodes a member of the ERF (ethylene response factor) subfamily B-1 of ERF/AP2 transcription factor family (ATERF-8). The protein contains one AP2 domain. There are 15 members in this subfamily including ATERF-3, ATERF-4, ATERF-7, and leafy petiole.

Table 5: Cluster 1: Some gene names and descriptions.

Gene Name	Description
At4g38250.1, At4g38260.1	Amino acid transporter family protein; similar to amino acid transporter family protein [Arabidopsis thaliana] (TAIR:AT2G42005.1); similar to amino acid transport protein (GB:AAB82307.1); similar to OSIGBa0158F05.8 [Oryza sativa (indica cultivar-group)] (GB:CAH66759.1); similar to OSJNBa0017B10.14 [Oryza sativa (japonica cultivar-group)] (GB:CAE03099.2); contains InterPro domain Amino acid/polyamine transporter II; (InterPro:IPR002422); contains InterPro domain Amino acid transporter, transmembrane; (InterPro:IPR013057) @ unknown protein; similar to unknown protein [Arabidopsis thaliana] (TAIR:AT1G20740.1); similar to H0409D10.8 [Oryza sativa (indica cultivar-group)] (GB:CAH66750.1); similar to Os09g0323500 [Oryza sativa (japonica cultivar-group)] (GB:NP_001062871.1); contains InterPro domain Protein of unknown function DUF833; (InterPro:IPR008551)
At5g22210.1, At5g22210.2	Unknown protein
At1g78270.1	UDP-glucose glucosyltransferase, putative; similar to UDP-glucoronosyl/UDP-glucosyl transferase family protein [Arabidopsis thaliana] (TAIR:AT1G22360.1); similar to transcription factor/ transferase, transferring glycosyl groups [Arabidopsis thaliana] (TAIR:AT1G22380.1); similar to UGT85A1 (UDP-glucosyl transferase 85A1), UDP-glycosyltransferase/ transferase, transferring glycosyl groups / transferase, transferring hexosyl groups [Arabidopsis thaliana] (TAIR:AT1G22400.1); similar to glycosyltransferase NTGT5b [Nicotiana tabacum] (GB:BAD93690.1); contains InterPro domain UDP-glucuronosyl/UDP-glucosyltransferase; (InterPro:IPR002213)
At2g29640.1	Josephin family protein; Identical to Josephin-like protein [Arabidopsis Thaliana] (GB:O82391); similar to josephin protein-related [Arabidopsis thaliana] (TAIR:AT1G07300.1); similar to unknown protein [Oryza sativa (japonica cultivar-group)] (GB:AAP06835.1); similar to Os03g0265200 [Oryza sativa (japonica cultivar-group)] (GB:NP_001049646.1); contains InterPro domain Machado-Joseph disease protein MJD; (InterPro:IPR006155)
At5g46190.1	KH domain-containing protein; similar to KH domain-containing protein [Arabidopsis thaliana] (TAIR:AT4G18375.2); similar to Os08g0200400 [Oryza sativa (japonica cultivar-group)] (GB:NP_001061211.1); similar to KH, type 1 [Medicago truncatula] (GB:ABE79454.1); contains InterPro domain KH; (InterPro:IPR004087); contains InterPro domain KH, type 1; (InterPro:IPR004088)
At5g41765.1	Unknown protein; similar to unknown protein [Arabidopsis thaliana] (TAIR:AT4G00232.1); contains InterPro domain Protein of unknown function DUF573; (InterPro:IPR007592)
At2g38050.1	DET2 (DE-ETIOLATED 2); Similar to mammalian steroid-5-alpha-reductase. Involved in the brassinolide biosynthetic pathway.

Table 6: Cluster 2: Some gene names and descriptions.

Gene Name	Description
At3g52500.1	Aspartyl protease family protein; similar to aspartyl protease family protein [Arabidopsis thaliana] (TAIR:AT4G16563.1); similar to aspartic protease [Fagopyrum esculentum] (GB:AAS48510.2); contains InterPro domain Peptidase A1, pepsin; (InterPro:IPR001461); contains InterPro domain Peptidase aspartic, catalytic; (InterPro:IPR009007)
At4g23820.1	Glycoside hydrolase family 28 protein / polygalacturonase (pectinase) family protein; similar to glycoside hydrolase family 28 protein / polygalacturonase (pectinase) family protein [Arabidopsis thaliana] (TAIR:AT5G41870.1); similar to Os05g0587000 [Oryza sativa (japonica cultivar-group)] (GB:NP_001056466.1); similar to Os02g0256100 [Oryza sativa (japonica cultivar-group)] (GB:NP_001046468.1); similar to putative polygalacturonase [Oryza sativa (japonica cultivar-group)] (GB:AAT44156.1); contains InterPro domain Virulence factor, pectin lyase fold; (InterPro:IPR011050); contains InterPro domain Glycoside hydrolase, family 28; (InterPro:IPR000743); contains InterPro domain Pectolytic enzyme, Pectin lyase fold; (InterPro:IPR012334)

Table 7: Cluster 3: Some gene names and descriptions.

Gene Name	Description
At3g15190.1	Chloroplast 30S ribosomal protein S20, putative; Identical to 30S ribosomal protein S20, chloroplast precursor (RPS20) [Arabidopsis Thaliana] (GB:Q9ASV6;GB:Q9LIL6); similar to Os01g0678600 [Oryza sativa (japonica cultivar-group)] (GB:NP_001043859.1); similar to ribosomal protein rpS20 [Bigelowiella natans] (GB:AAP79183.1); contains InterPro domain Ribosomal protein S20; (InterPro:IPR002583); contains InterPro domain Ribosomal protein S20p; (InterPro:IPR010013)
At1g15290.1	Binding; similar to binding [Arabidopsis thaliana] (TAIR:AT4G28080.1); similar to tetratricopeptide repeat (TPR)-containing protein [Arabidopsis thaliana] (TAIR:AT1G01320.1); similar to putative tetratricopeptide repeat(TPR)-containing protein [Oryza sativa (japonica cultivar-group)] (GB:BAC84544.1); similar to TPR repeat [Medicago truncatula] (GB:ABE77904.1); similar to H0811D08.1 [Oryza sativa (indica cultivar-group)] (GB:CAJ86110.1); contains InterPro domain Tetratricopeptide region; (InterPro:IPR013026); contains InterPro domain Tetratricopeptide TPR_1; (InterPro:IPR001440); contains InterPro domain Tetratricopeptide TPR_2; (InterPro:IPR013105); contains InterPro domain Tetratricopeptide-like helical; (InterPro:IPR011990)

Table 8: Cluster 4: Some gene names and descriptions.

Gene Name	Description
At5g36170.1, At5g36170.2, At5g36170.3	HCF109 (HIGH CHLOROPHYLL FLUORESCENT 109); translation release factor; Required for normal processing of polycistronic plastidial transcripts
At1g72310.1	ATL3 (Arabidopsis Tx́icos en Levadura 3); protein binding / zinc ion binding; Encodes a putative RING-H2 zinc finger protein ATL3 (ATL3).

Table 9: Cluster 5: Some gene names and descriptions.

Gene Name	Description
At1g47900.1	Unknown protein; similar to unknown protein [Arabidopsis thaliana] (TAIR:AT1G19835.1); similar to Putative myosin-like protein [Oryza sativa (japonica cultivar-group)] (GB:AAL77142.1); similar to Os03g0246500 [Oryza sativa (japonica cultivar-group)] (GB:NP_001049544.1); contains InterPro domain Protein of unknown function DUF869, plant; (InterPro:IPR008587)

Table 10: Cluster 6: Some gene names and descriptions.

Gene Name	Description
At2g39725.1, At2g39725.2	Complex 1 family protein / LVR family protein; similar to Os08g0278600 [Oryza sativa (japonica cultivar-group)] (GB:NP_001061438.1); similar to unknown protein [Oryza sativa (japonica cultivar-group)] (GB:BAC99750.1); contains InterPro domain Complex 1 LYR protein; (InterPro:IPR008011)
At5g07410.1	Pectinesterase family protein; similar to ATPPME1, pectinesterase [Arabidopsis thaliana] (TAIR:AT1G69940.1); similar to pectin methylesterase allergenic protein [Salsola kali] (GB:AAX11262.1); contains InterPro domain Virulence factor, pectin lyase fold; (InterPro:IPR011050); contains InterPro domain Pectinesterase; (InterPro:IPR000070)

Table 11: Cluster 7: Some gene names and descriptions.

Gene Name	Description
At1g59670.1	ATGSTU15 (Arabidopsis thaliana Glutathione S-transferase (class tau) 15); glutathione transferase; Encodes glutathione transferase belonging to the tau class of GSTs. Naming convention according to Wagner et al. (2002).
At1g32960.1	Subtilase family protein; similar to subtilase family protein [Arabidopsis thaliana] (TAIR:AT1G32950.1); similar to subtilase family protein [Arabidopsis thaliana] (TAIR:AT4G10540.1); similar to subtilase family protein [Arabidopsis thaliana] (TAIR:AT1G32940.1); similar to Os09g0530800 [Oryza sativa (japonica cultivar-group)] (GB:NP_001063751.1); similar to Protease-associated PA; Proteinase inhibitor I9, subtilisin propeptide [Medicago truncatula] (GB:ABE90461.1); contains InterPro domain Protease-associated PA; (InterPro:IPR003137); contains InterPro domain Peptidase S8 and S53, subtilisin, kexin, sedolisin; (InterPro:IPR000209); contains InterPro domain Proteinase inhibitor I9, subtilisin propeptide; (InterPro:IPR010259); contains InterPro domain Proteinase inhibitor, propeptide; (InterPro:IPR009020)
At2g39440.1	Unknown protein; similar to unknown protein [Arabidopsis thaliana] (TAIR:AT1G61280.1); similar to hypothetical protein MtrDRAFT_AC126784g11v2 [Medicago truncatula] (GB:ABE94681.1); contains InterPro domain PIG-P; (InterPro:IPR013717)
At3g54140.1	Proton-dependent oligopeptide transport (POT) family protein; similar to ATPTR2-B (NITRATE TRANSPORTER 1), transporter [Arabidopsis thaliana] (TAIR:AT2G02040.1); similar to proton-dependent oligopeptide transport (POT) family protein [Arabidopsis thaliana] (TAIR:AT1G62200.1); similar to proton-dependent oligopeptide transport (POT) family protein [Arabidopsis thaliana] (TAIR:AT5G01180.1); similar to LeOPT1 [Lycopersicon esculentum] (GB:AAD01600.1); similar to putative peptide transport protein [Oryza sativa (japonica cultivar-group)] (GB:BAD31819.1); similar to peptide transporter [Hordeum vulgare] (GB:AAC32034.1); contains InterPro domain TGF-beta receptor, type I/II extracellular region; (InterPro:IPR000109)

Table 12: Cluster 8: Some gene names and descriptions.

Gene Name	Description
At3g55470.1, At3g55470.2	C2 domain-containing protein; similar to C2 domain-containing protein [Arabidopsis thaliana] (TAIR:AT1G63220.1); similar to Os-FIERG2 gene product [Oryza sativa] (GB:AAC04628.1); contains InterPro domain C2; (InterPro:IPR000008); contains InterPro domain C2 calcium/lipid-binding region, CaLB; (InterPro:IPR008973)
At2g35070.1	Unknown protein; similar to unknown protein [Arabidopsis thaliana] (TAIR:AT2G35090.1); similar to conserved hypothetical protein [Medicago truncatula] (GB:ABE89621.1); contains domain UNCHARACTERIZED (PTHR14360)
At1g27300.1	Unknown protein; similar to Os02g0509600 [Oryza sativa (japonica cultivar-group)] (GB:NP_001046928.1)

Table 13: Cluster 9: Some gene names and descriptions.
