# Supplementary material for: Listen to Genes: Dealing with Microarray Data in the Frequency Domain
Source: PLoS One. 2009 Apr 6;4(4):e5098. doi: 10.1371/journal.pone.0005098 (PMC3383793; doi:10.1371/journal.pone.0005098)
Supplement: Text S5 — Complex Interactions (0.45 MB PDF) [file pone.0005098.s005.pdf]

Text S5: Complex Interactions

Introduction

A network structure carefully inferred from experimental data can provide us with critical information about the underlying system of investigation and is an important topic in system biology. For example, high-throughput data from gene, metabolic, signaling or transcriptional regulatory networks, contain information about thousands of genes and products thereof. For such complex networks, complex (group) interactions occur since the nodes (genes, proteins and substances) may work cooperatively or competitively to achieve a task. Complex interactions are inherently multivariate in nature and differ from pairwise bivariate interactions. Two variables may not interact with the third variable on a pairwise basis but a combination of them may result in an interaction with the third one. Alternatively, two negatively correlated variables when combined together can show no interaction with a third variable, even though the individual pairwise interactions between them were existing.

As another example, consider the chemical reaction from S to P. An enzyme E acts as a catalyst from S to P, but not from P to S. Hence, from observed data to find that E is a cause of (S, P), but not (P, S) is obviously an interesting and challenging task (see Fig. 1). To fully understand the properties of a network, whether it is a gene, a substance, a protein or a neuronal network, it is therefore of essential importance to consider complex interactions.

This issue has been experimentally realized and tested intensively. For example, LOF (loss of function) experiments are performed for double, triple and quadruple mutations. Two computational approaches to explore the experimental data and recover the interactions between units in the literature are Bayesian network and Granger causality. However, to the best of our knowledge, no systematical approach has been developed to take this issue into account. Here we adopt the Granger causality approach. The concept of the Granger causality originally introduced by Wiener [8] and formulated by Granger [4] has played a considerably important role in investigating the relationship among stationary time series. Specifically, given two time series, if the variance of the prediction error for the second

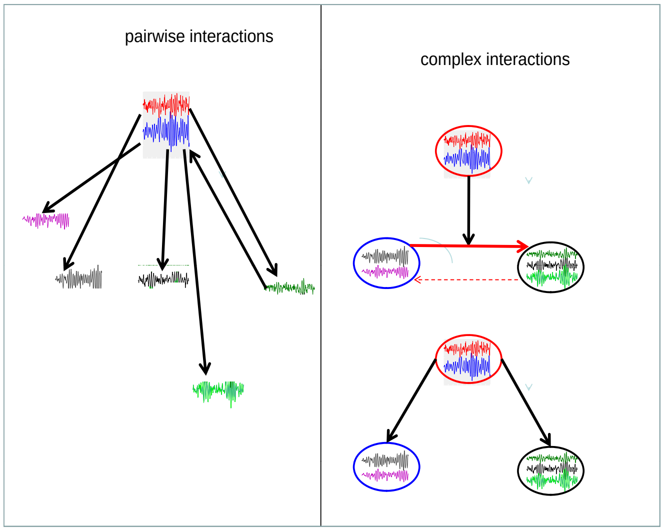

Figure 1: A schematic plot of the complex interactions. Each time trace (node) is the activity of a gene, protein, substance etc. A circle is a complex comprising of nodes. Left panel is the interactions among nodes. Right panel, The top complex could only exert its influence on the rate between two complexes (top), or on the complexes themselves (bottom).

time series at the present time is reduced by including past measurements from the first time series in the (non)linear regression model, then the first time series can be said to cause the second time series. Geweke's decomposition of a vector autoregressive process [2] led to a set of causality measures which have a spectral representation and make the interpretation more informative and useful.

In this paper, we first extend the pairwise Granger causality and the partial Granger causality (proposed by us in [5]) to the complex Granger causality, both in time and frequency domain. We then apply our approach to synthesized and experimental data to validate the efficiency of our approach. In the synthesized data, we first demonstrate that our complex Granger causality can reliably detect the pairwise complex interactions, both in time and frequency domain. Purely on terms of the data, complex interactions can arise even when there is no interactions between individual nodes. In order to deal with the interactions between multi-complexes, we then extend the pairwise complex Granger causality to partial complex Granger causality. The partial complex Granger causality is then applied

to two biological datasets: a microarray gene-expression dataset for Arabidopsis leaf and a metabolic reaction dataset. In the microarray data, we concentrate on a circadian circuit of 7 genes. In agreement with the experimental findings, we show that gene GI has no interaction with the remaining 6 genes when only the interactions among individual genes are taken into account. GI clearly plays a role when complex interactions are considered. In the metabolic reaction chain, our approach identifies that the enzyme E definitely acts on a reaction rate from the substance S to the substance P_1, but not on the substances themselves. Finally a theoretical result is presented to illustrate the impact of cooperative and competitive relationship between nodes on the complex Granger causality.

1 Methods

1.1 Pairwise Complex Granger causality

1.1.1 Time Domain Formulation

Consider two multivariate stationary time series $\vec{X}_t$ and $\vec{Y}_t$ with k and l dimensions respectively. Individually, under fairly general conditions, each time series has the following vector autoregressive representation

$$\left\{ \begin{array}{lcl} \vec{X}_t & = & \sum\limits_{i=1}^{\infty} A_{1i}\vec{X}_{t-i} + \overrightarrow{\epsilon_{1t}} \\ \vec{Y}_t & = & \sum\limits_{i=1}^{\infty} B_{1i}\vec{Y}_{t-i} + \overrightarrow{\epsilon_{2t}} \end{array} \right. \tag{13}$$

where $\overrightarrow{\epsilon_{it}}, i = 1, 2$ are normally distributed random vectors with k and l dimensions. Their contemporaneous covariance matrix are Γ_{xx} and Γ_{yy} with the traces being denoted by T_x and T_y respectively. The value of T_x is non-negative and equals the summation of all eigenvalues of Γ_{xx}, which measures the accuracy of the autoregressive prediction of X based on its previous values, whereas the value of T_y represents the accuracy of predicting present value of $\vec{Y}$ based on previous values of $\vec{Y}$.

Jointly, they are represented as

$$\left\{ \begin{array}{lcl} \vec{X}_t & = & \sum\limits_{i=1}^{\infty} A_{2i}\vec{X}_{t-i} + \sum\limits_{i=1}^{\infty} B_{2i}\vec{Y}_{t-i} + \overrightarrow{\epsilon_{3t}} \\ \vec{Y}_t & = & \sum\limits_{i=1}^{\infty} C_{2i}\vec{X}_{t-i} + \sum\limits_{i=1}^{\infty} D_{2i}\vec{Y}_{t-i} + \overrightarrow{\epsilon_{4t}} \end{array} \right. \tag{14}$$

where the noise terms are uncorrelated over time and their contemporaneous covariance matrix is

$$\Sigma = \begin{pmatrix} \Sigma_{xx} & \Sigma_{xy} \\ \Sigma_{yx} & \Sigma_{yy} \end{pmatrix} \tag{15}$$

The submatrix are defined as $\Sigma_{xx} = \text{var}(\overrightarrow{\epsilon_{3t}}), \Sigma_{xy} = \text{cov}(\overrightarrow{\epsilon_{3t}}, \overrightarrow{\epsilon_{4t}}), \Sigma_{yx} = \text{cov}(\overrightarrow{\epsilon_{4t}}, \overrightarrow{\epsilon_{3t}}), \Sigma_{yy} = \text{var}(\overrightarrow{\epsilon_{4t}})$.

If X_t and Y_t are independent, the coefficient matrix B_{2i} and C_{2i} are zero, $\Gamma_{xx} = \Sigma_{xx}, \Gamma_{yy} = \Sigma_{yy}$, $\Sigma_{xy} = \Sigma_{yx}^{'} = \mathbf{0}$. The trace of Σ_{xx} and Σ_{yy} is denoted by T_{xy} and T_{yx} respectively. Consider eq. (14), the value of T_{xy} represents the accuracy of predicting present value of $\vec{X}$ based on previous values of both $\vec{X}$ and $\vec{Y}$. According to the causality definition of Granger, if the prediction of one time series is improved by incorporating past information of the second time series, then the second time series causes the first process. We extend them to multiple dimensional cases. If the trace of prediction error for the first multiple time series is reduced by the inclusion of past histories of the second multiple time series, then a causal relation from the second multiple time series to the first multiple time series exists. We quantify this causal influence by

$$F_{\vec{Y}\to\vec{X}} = \ln(T_x/T_{xy}) \tag{16}$$

It is clear that $F_{\vec{Y}\to\vec{X}} = 0$ when there is no causal influence from $\vec{X}$ to $\vec{Y}$ otherwise $F_{\vec{X}\to\vec{Y}} > 0$.

1.1.2 Frequency Domain Formulation

Time series contain oscillatory aspects in specific frequency bands. It is thus desirable to to have a spectral representation of causal influence. We then consider the frequency domain formulation of complex Granger causality. Rewrite eqs. (14) in terms of the lag operator

$$\begin{pmatrix} A_2(L) & B_2(L) \\ C_2(L) & D_2(L) \end{pmatrix} \begin{pmatrix} \vec{X}_t \\ \vec{Y}_t \end{pmatrix} = \begin{pmatrix} \overrightarrow{\epsilon_{3t}} \\ \overrightarrow{\epsilon_{4t}} \end{pmatrix} \tag{17}$$

where $A_2(0) = I_k, B_2(0) = \mathbf{0}, C_2(0) = \mathbf{0}, d_2(0) = I_l$. Fourier transforming both sides of eqs.(17) leads to

$$\begin{pmatrix} A_2(\omega) & B_2(\omega) \\ C_2(\omega) & D_2(\omega) \end{pmatrix} \begin{pmatrix} X(\omega) \\ Y(\omega) \end{pmatrix} = \begin{pmatrix} E_x(\omega) \\ E_y(\omega) \end{pmatrix} \tag{18}$$

where the components of the coefficient matrix are

$$A_2(\omega) = I_k - \sum_{i=1}^{\infty} A_{2i} e^{-i\omega j}, \ \ B_2(\omega) = -\sum_{i=1}^{\infty} B_{2i} e^{-i\omega j},$$

$$C_2(\omega) = -\sum_{i=1}^{\infty} C_{2i} e^{-i\omega j}, \ \ D_2(\omega) = I_l - \sum_{i=1}^{\infty} D_{2i} e^{-i\omega j},$$

Recasting eq.(18) into the transfer function format we obtain

$$\begin{pmatrix} X(\omega) \\ Y(\omega) \end{pmatrix} = \begin{pmatrix} H_{xx}(\omega) & H_{xy}(\omega) \\ H_{yx}(\omega) & H_{yy}(\omega) \end{pmatrix} \begin{pmatrix} E_x(\omega) \\ E_y(\omega) \end{pmatrix} \tag{19}$$

the components of $\mathbf{H}(\omega)$ are

$$H_{yy}(\omega) = (D_2(\omega) - C_2(\omega)A_2(\omega)^{-1}B_2(\omega))^{-1}$$

$$H_{xy}(\omega) = -A_2(\omega)^{-1}B_2(\omega)H_{yy}(\omega)$$

$$H_{yx}(\omega) = -H_{yy}(\omega)C_2(\omega)A_2(\omega)^{-1}$$

$$H_{xx}(\omega) = A_2(\omega)^{-1} - H_{xy}(\omega)C_2(\omega)A_2(\omega)^{-1}$$

After proper ensemble averaging we have the spectral matrix

$$S(\omega) = \mathbf{H}(\omega)\Sigma\mathbf{H}^*(\omega)$$

where * denotes the complex conjugate and matrix transpose, and Σ is defined in eq. (15).

To obtain the frequency decomposition of the time domain causality defined in the previous section, we look at the auto-spectrum of X_t

$$S_{xx}(\omega) = H_{xx}(\omega)\Sigma_{xx}H^*_{xx}(\omega) + H_{xx}(\omega)\Sigma_{xy}H^*_{xy}(\omega) + H_{xy}(\omega)\Sigma_{yx}H^*_{xx}(\omega) + H_{xy}(\omega)\Sigma_{yy}H^*_{xy}(\omega) \tag{20}$$

Note that the value of diagonal of $S_{xx}(\omega)$ is real numbers, the trace of both sides can be represented as

$$\begin{aligned} \text{tr}(S_{xx}(\omega)) &= \text{tr}(H_{xx}(\omega)\Sigma_{xx}H^*_{xx}(\omega)) + \text{tr}(H_{xx}(\omega)\Sigma_{xy}H^*_{xy}(\omega) + H_{xy}(\omega)\Sigma_{yx}H^*_{xx}(\omega)) \\ &\quad + \text{tr}(H_{xy}(\omega)\Sigma_{yy}H^*_{xy}(\omega)) \end{aligned} \tag{21}$$

We first consider a simple case of $\Sigma_{xy} = \mathbf{0}$. The second term on the right side of eq.(21) is zero. We have

$$\text{tr}(S_{xx}(\omega)) = \text{tr}(H_{xx}(\omega)\Sigma_{xx}H^*_{xx}(\omega)) + \text{tr}(H_{xy}(\omega)\Sigma_{yy}H^*_{xy}(\omega)) \tag{22}$$

which implies that the spectrum of X_t has two terms, the first term, viewed as the intrinsic part, involves only the noise term that drives the X_t time series. The second term, viewed as the causal part, involves only the noise term that drives Y_t.

When $\Sigma_{xy} \neq \mathbf{0}$, we can normalize eq. (18) by multiplying the following matrix

$$P = \begin{pmatrix} I_k & \mathbf{0} \\ -\Sigma_{yx}\Sigma_{xx}^{-1} & I_l \end{pmatrix} \tag{23}$$

to both sides of eq.(18). The results is

$$\begin{pmatrix} A_2(\omega) & B_2(\omega) \\ C_3(\omega) & D_3(\omega) \end{pmatrix} \begin{pmatrix} X(\omega) \\ Y(\omega) \end{pmatrix} = \begin{pmatrix} E_x(\omega) \\ \tilde{E}_y(\omega) \end{pmatrix} \tag{24}$$

where $C_3(\omega) = C_2(\omega) - \Sigma_{yx}\Sigma_{xx}^{-1}A_2(\omega), D_3(\omega) = D_2(\omega) - \Sigma_{yx}\Sigma_{xx}^{-1}B_2(\omega), \tilde{E}_y(\omega) = E_y(\omega) - \Sigma_{yx}\Sigma_{xx}^{-1}E_x(\omega)$. From the construction it is easy to see that E_x and $\tilde{E}_y$ are uncorrelated. The variance of the noise term for the normalized Y_t equation is $\Sigma_{yy} - \Sigma_{yx}\Sigma_{xx}^{-1}\Sigma_{xy}$. The new transfer function $\tilde{\mathbf{H}}(\omega)$ for eq.(24) is the inverse of the new coefficient matrix

$$\tilde{\mathbf{H}}(\omega) = \begin{pmatrix} \tilde{H}_{xx}(\omega) & \tilde{H}_{xy}(\omega) \\ \tilde{H}_{yx}(\omega) & \tilde{H}_{yy}(\omega) \end{pmatrix} \tag{25}$$

where

$$\tilde{H}_{xx}(\omega) = H_{xx}(\omega) + H_{xy}(\omega)\Sigma_{yx}\Sigma_{xx}^{-1}, \quad \tilde{H}_{xy}(\omega) = H_{xy}(\omega)$$
$$\tilde{H}_{yx}(\omega) = H_{yx}(\omega) + H_{yy}(\omega)\Sigma_{yx}\Sigma_{xx}^{-1}, \quad \tilde{H}_{yy}(\omega) = H_{yy}(\omega)$$

Note that E_x and $\tilde{E}_y$ are uncorrelated, following the same steps of eq.(22), the spectrum of X_t is found to be

$$S_{xx}(\omega) = \tilde{H}_{xx}(\omega)\Sigma_{xx}\tilde{H}^*_{xx}(\omega) + \tilde{H}_{xy}(\omega)(\Sigma_{yy} - \Sigma_{yx}\Sigma_{xx}^{-1}\Sigma_{xy})\tilde{H}^*_{xy}(\omega) \tag{26}$$

The trace of both sides can be represented as

$$\text{tr}(S_{xx}(\omega)) = \text{tr}(\tilde{H}_{xx}(\omega)\Sigma_{xx}\tilde{H}^*_{xx}(\omega)) + \text{tr}(\tilde{H}_{xy}(\omega)(\Sigma_{yy} - \Sigma_{yx}\Sigma_{xx}^{-1}\Sigma_{xy})\tilde{H}^*_{xy}(\omega)) \tag{27}$$

Here he first term is interpreted as the intrinsic power and the second term as the causal power of X_t due to Y_t. We define the causal influence from Y_t to X_t at frequency ω as

$$f_{Y\to X}(\omega) = \ln \frac{\text{tr}(S_{xx}(\omega))}{\text{tr}(\tilde{H}_{xx}(\omega)\Sigma_{xx}\tilde{H}^*_{xx}(\omega))} \tag{28}$$

1.2 Partial Complex Granger causality

1.2.1 Time Domain Formulation

Consider three multiple stationary time series X_t, Y_t and Z_t with k, l and m dimensions respectively. We first consider the relationship from Y_t to X_t on condition of Z_t. The joint autoregressive representation for X_t and Z_t can be written as

$$\begin{cases} X_t &= \sum_{i=1}^{\infty} a_{1i}X_{t-i} + \sum_{i=1}^{\infty} c_{1i}Z_{t-i} + \overrightarrow{\epsilon_{1t}} \\ Z_t &= \sum_{i=1}^{\infty} b_{1i}Z_{t-i} + \sum_{i=1}^{\infty} d_{1i}X_{t-i} + \overrightarrow{\epsilon_{2t}} \end{cases} \tag{29}$$

The noise covariance matrix for the system can be represented as

$$\Gamma = \begin{pmatrix} \text{var}(\overrightarrow{\epsilon_{1t}}) & \text{cov}(\overrightarrow{\epsilon_{1t}}, \overrightarrow{\epsilon_{2t}}) \\ \text{cov}(\overrightarrow{\epsilon_{2t}}, \overrightarrow{\epsilon_{1t}}) & \text{var}(\overrightarrow{\epsilon_{2t}}) \end{pmatrix} = \begin{pmatrix} \Gamma_{xx} & \Gamma_{xz} \\ \Gamma_{zx} & \Gamma_{zz} \end{pmatrix}$$

where var and cov represent variance and co-variance respectively. Extending this representation, the vector autoregressive representation for a system involving three time series X_t, Y_t and Z_t can be written in the following way.

$$\begin{cases} X_t &= \sum_{i=1}^{\infty} a_{2i}X_{t-i} + \sum_{i=1}^{\infty} b_{2i}Y_{t-i} + \sum_{i=1}^{\infty} c_{2i}Z_{t-i} + \overrightarrow{\epsilon_{3t}} \\ Y_t &= \sum_{i=1}^{\infty} d_{2i}X_{t-i} + \sum_{i=1}^{\infty} e_{2i}Y_{t-i} + \sum_{i=1}^{\infty} f_{2i}Z_{t-i} + \overrightarrow{\epsilon_{4t}} \\ Z_t &= \sum_{i=1}^{\infty} g_{2i}X_{t-i} + \sum_{i=1}^{\infty} h_{2i}Y_{t-i} + \sum_{i=1}^{\infty} k_{2i}Z_{t-i} + \overrightarrow{\epsilon_{5t}} \end{cases} \tag{30}$$

The noise covariance matrix for the above system can be represented as

$$\Sigma = \begin{pmatrix} \text{var}(\overrightarrow{\epsilon_{3t}}) & \text{cov}(\overrightarrow{\epsilon_{3t}}, \overrightarrow{\epsilon_{4t}}) & \text{cov}(\overrightarrow{\epsilon_{3t}}, \overrightarrow{\epsilon_{5t}}) \\ \text{cov}(\overrightarrow{\epsilon_{4t}}, \overrightarrow{\epsilon_{3t}}) & \text{var}(\overrightarrow{\epsilon_{4t}}) & \text{cov}(\overrightarrow{\epsilon_{4t}}, \overrightarrow{\epsilon_{5t}}) \\ \text{cov}(\overrightarrow{\epsilon_{5t}}, \overrightarrow{\epsilon_{3t}}) & \text{cov}(\overrightarrow{\epsilon_{5t}}, \overrightarrow{\epsilon_{4t}}) & \text{var}(\overrightarrow{\epsilon_{5t}}) \end{pmatrix} = \begin{pmatrix} \Sigma_{xx} & \Sigma_{xy} & \Sigma_{xz} \\ \Sigma_{yx} & \Sigma_{yy} & \Sigma_{yz} \\ \Sigma_{zx} & \Sigma_{zy} & \Sigma_{zz} \end{pmatrix}$$

where $\overrightarrow{\epsilon_{it}}, i = 1, \cdots, 5$ are the prediction error uncorrelated over time. The conditional variance $\Gamma_{xx} - \Gamma_{xz}\Gamma_{zz}^{-1}\Gamma_{zx}$ measures the accuracy of the autoregressive prediction of X

based on its previous values conditioned on Z whereas the conditional variance $\Sigma_{xx} - \Sigma_{xz}\Sigma_{zz}^{-1}\Sigma_{zx}$ measures the accuracy of the autoregressive prediction of X based on its previous values of both X and Y conditioned on Z. The trace of matrix $\Gamma_{xx} - \Gamma_{xz}\Gamma_{zz}^{-1}\Gamma_{zx}$ and matrix $\Sigma_{xx} - \Sigma_{xz}\Sigma_{zz}^{-1}\Sigma_{zx}$ are denoted by $T_{x|z}$ and $T_{xy|z}$ respectively. We define the partial Granger causality from vector Y to vector X conditioned on vector Z to be

$$F_{Y\to X|Z} = \ln\left(\frac{T_{x|z}}{T_{xy|z}}\right) \tag{31}$$

1.2.2 Frequency Domain Formulation

To drive the spectral decomposition of the time domain partial Granger causality, we first multiply the matrix

$$P_1 = \begin{pmatrix} I_k & -\Gamma_{xz}\Gamma_{zz}^{-1} \\ \mathbf{0} & I_m \end{pmatrix} \tag{32}$$

to both sides of eq. (29). The normalized equations are represented as:

$$\begin{pmatrix} D_{11}(L) & D_{12}(L) \\ D_{21}(L) & D_{22}(L) \end{pmatrix} \begin{pmatrix} X_t \\ Z_t \end{pmatrix} = \begin{pmatrix} \tilde{X}_t \\ \tilde{Z}_t \end{pmatrix} \tag{33}$$

with $D_{11}(0) = I_k, D_{22}(0) = I_m, D_{21}(0) = \mathbf{0}, \text{cov}(\tilde{X}_t, \tilde{Z}_t) = 0$, we note that $\text{var}(\tilde{X}_t) = \Gamma_{xx} - \Gamma_{xz}\Gamma_{zz}^{-1}\Gamma_{zx}, \text{var}(\tilde{Z}_t) = \Gamma_{zz}$. For eq. (30), we also multiply the matrix

$$P = P_3 \cdot P_2 \tag{34}$$

where

$$P_2 = \begin{pmatrix} I_k & \mathbf{0} & -\Sigma_{xz}\Sigma_{zz}^{-1} \\ \mathbf{0} & I_l & -\Sigma_{yz}\Sigma_{zz}^{-1} \\ \mathbf{0} & \mathbf{0} & I_m \end{pmatrix} \tag{35}$$

and

$$P_3 = \begin{pmatrix} I_k & \mathbf{0} & \mathbf{0} \\ -(\Sigma_{yx} - \Sigma_{yz}\Sigma_{zz}^{-1}\Sigma_{zx})(\Sigma_{xx} - \Sigma_{xz}\Sigma_{zz}^{-1}\Sigma_{zx})^{-1} & I_l & \mathbf{0} \\ \mathbf{0} & \mathbf{0} & I_m \end{pmatrix} \tag{36}$$

to both sides of eq.(30). The normalized equation of eq. (30) becomes

$$\begin{pmatrix} B_{11}(L) & B_{12}(L) & B_{13}(L) \\ B_{21}(L) & B_{22}(L) & B_{23}(L) \\ B_{31}(L) & B_{32}(L) & B_{33}(L) \end{pmatrix} \begin{pmatrix} X_t \\ Y_t \\ Z_t \end{pmatrix} = \begin{pmatrix} \epsilon_{xt} \\ \epsilon_{yt} \\ \epsilon_{zt} \end{pmatrix} \tag{37}$$

where $\epsilon_{xt}, \epsilon_{yt}, \epsilon_{zt}$ are independent, and their variances being $\hat{\Sigma}_{xx}$, $\hat{\Sigma}_{yy}$ and $\hat{\Sigma}_{zz}$ with

$$\begin{cases} \hat{\Sigma}_{zz} &= \Sigma_{zz} \\ \hat{\Sigma}_{xx} &= \Sigma_{xx} - \Sigma_{xz}\Sigma_{zz}^{-1}\Sigma_{zx} \\ \hat{\Sigma}_{yy} &= \Sigma_{yy} - \Sigma_{yz}\Sigma_{zz}^{-1}\Sigma_{zy} - \dfrac{(\Sigma_{yx} - \Sigma_{yz}\Sigma_{zz}^{-1}\Sigma_{zx})(\Sigma_{xy} - \Sigma_{xz}\Sigma_{zz}^{-1}\Sigma_{zy})}{(\Sigma_{yy} - \Sigma_{yz}\Sigma_{zz}^{-1}\Sigma_{zy})} \end{cases}$$

After Fourier transforming eq. (33) and eq. (37), we can rewrite these equations in the following way:

$$\begin{pmatrix} X(\omega) \\ Z(\omega) \end{pmatrix} = \begin{pmatrix} G_{xx}(\omega) & G_{xz}(\omega) \\ G_{zx}(\omega) & G_{zz}(\omega) \end{pmatrix} \begin{pmatrix} \tilde{X}(\omega) \\ \tilde{Z}(\omega) \end{pmatrix} \tag{38}$$

and

$$\begin{pmatrix} X(\omega) \\ Y(\omega) \\ Z(\omega) \end{pmatrix} = \begin{pmatrix} H_{xx}(\omega) & H_{xy}(\omega) & H_{xz}(\omega) \\ H_{yx}(\omega) & H_{yy}(\omega) & H_{yz}(\omega) \\ H_{zx}(\omega) & H_{zy}(\omega) & H_{zz}(\omega) \end{pmatrix} \begin{pmatrix} E_x(\omega) \\ E_y(\omega) \\ E_z(\omega) \end{pmatrix} \tag{39}$$

Note that $X(\omega)$ and $Z(\omega)$ from eq. (38) are identical with that from eq. (39), we thus have

$$\begin{aligned} \begin{pmatrix} \tilde{X}(\omega) \\ Y(\omega) \\ \tilde{Z}(\omega) \end{pmatrix} &= \begin{pmatrix} G_{xx}(\omega) & 0 & G_{xz}(\omega) \\ 0 & 1 & 0 \\ G_{zx}(\omega) & 0 & G_{zz}(\omega) \end{pmatrix}^{-1} \begin{pmatrix} H_{xx}(\omega) & H_{xy}(\omega) & H_{xz}(\omega) \\ H_{yx}(\omega) & H_{yy}(\omega) & H_{yz}(\omega) \\ H_{zx}(\omega) & H_{zy}(\omega) & H_{zz}(\omega) \end{pmatrix} \begin{pmatrix} E_x(\omega) \\ E_y(\omega) \\ E_z(\omega) \end{pmatrix} \\ &= \begin{pmatrix} Q_{xx}(\omega) & Q_{xy}(\omega) & Q_{xz}(\omega) \\ Q_{yx}(\omega) & Q_{yy}(\omega) & Q_{yz}(\omega) \\ Q_{zx}(\omega) & Q_{zy}(\omega) & Q_{zz}(\omega) \end{pmatrix} \begin{pmatrix} E_x(\omega) \\ E_y(\omega) \\ E_z(\omega) \end{pmatrix} \end{aligned} \tag{40}$$

where $\mathbf{Q}(\omega) = \mathbf{G}^{-1}(\omega)\mathbf{H}(\omega)$. Now the power spectrum of $\tilde{X}$ is

$$S_{\tilde{x}\tilde{x}}(\omega) = Q_{xx}(\omega)\hat{\Sigma}_{xx}Q_{xx}^*(\omega) + Q_{xy}(\omega)\hat{\Sigma}_{yy}Q_{xy}^*(\omega) + Q_{xz}(\omega)\hat{\Sigma}_{zz}Q_{xz}^*(\omega) \tag{41}$$

The trace of both sides of eq. (41) can be represented as

$$\text{tr}(S_{\tilde{x}\tilde{x}}(\omega)) = \text{tr}(Q_{xx}(\omega)\hat{\Sigma}_{xx}Q_{xx}^*(\omega)) + \text{tr}(Q_{xy}(\omega)\hat{\Sigma}_{yy}Q_{xy}^*(\omega)) + \text{tr}(Q_{xz}(\omega)\hat{\Sigma}_{zz}Q_{xz}^*(\omega)) \tag{42}$$

Note that $\hat{\Sigma}_{xx} = \Sigma_{xx} - \Sigma_{xz}\Sigma_{zz}^{-1}\Sigma_{zx}$, the first term can be thought of as the intrinsic power eliminating exogenous inputs and latent variables and the remaining two terms as the combined causal influence from Y on the mediate of Z. This interpretation leads immediately to the definition

$$f_{Y\to X|Z}(\omega) = \ln \frac{\text{tr}(S_{\tilde{x}\tilde{x}}(\omega))}{\text{tr}(Q_{xx}(\omega)\hat{\Sigma}_{xx}Q_{xx}^{*}(\omega))} \tag{43}$$

Note that according to eq. (31), the variance of X^* equals to $\Gamma_{xx} - \Gamma_{xz}\Gamma_{zz}^{-1}\Gamma_{zx}$, By the Kolmogrov formula [3] for spectral decompositions and under some mild conditions, the Granger causality measures in the frequency domain and in the time domain satisfy

$$F_{Y\to X|Z} = \frac{1}{2\pi}\int_{-\pi}^{\pi} f_{Y\to X|Z}(\omega)d\omega \tag{44}$$

2 Results

2.1 Synthesized data: pairwise complex interaction

Example 1 Suppose that two simultaneously generated multiple time series are defined by the equations

$$\begin{cases} X_t &= A_{11}X_{t-1} + A_{12}X_{t-2} + \overrightarrow{\epsilon_{1t}} \\ Y_t &= A_{21}X_{t-1} + A_{22}X_{t-2} + B_{21}Y_{t-1} + \overrightarrow{\epsilon_{2t}} \end{cases}$$

where $X_t = (x_{1t}, x_{2t})^T$ is a 2 dimensional vector, $Y_t = (x_{3t}, x_{4t}, x_{5t})^T$ is a 3 dimensional vector, $\overrightarrow{\epsilon_{1t}}, \overrightarrow{\epsilon_{2t}}$ are normally distributed random vectors. The coefficient matrices are

$$A_{11} = \begin{pmatrix} 0.95\sqrt{2} & 0 \\ 0 & 0 \end{pmatrix}, A_{12} = \begin{pmatrix} -0.9025 & 0 \\ -0.5 & 0 \end{pmatrix}, A_{21} = \begin{pmatrix} 0.1 & 0 \\ 0 & 0 \\ 0 & 0 \end{pmatrix}, A_{22} = \begin{pmatrix} 0 & 0.4 \\ 0 & 0 \\ 0 & 0 \end{pmatrix},$$

$$B_{21} = \begin{pmatrix} 0 & 0 & 0 \\ -0.5 & 0.25\sqrt{2} & 0.25\sqrt{2} \\ 0 & -0.25\sqrt{2} & 0.25\sqrt{2} \end{pmatrix}$$

We perform a simulation of this system by generating a data set of 2000 data points with a sample rate of 200 Hz. The traces of the two vectors X_t and Y_t are plotted in Fig. 2 (**A**) (inside ovals). It is clear that X_t is a direct source to Y_t while Y_t is not a direct

source to X_t, the true structure is plotted in Fig 2 (**A**). Fig 2 (**B**) presents a comparison between the time domain pairwise complex Granger causality and the frequency domain pairwise complex Granger causality (see Fig 2 (**C**) for details). Blue dots are the values of the pairwise complex Granger causality calculated in the time domain. Red dots are the summation (integration) of the pairwise complex Granger causality for frequencies in the range of $[-\pi, \pi]$. As expected, Fig 2 (**C**) demonstrates that the decomposition in the frequency domain fits very well with the pairwise Granger causality in the time domain. Fig 2 (**C**) is the results obtained in the frequency domain. It is easy to see that there is direct causal drives from X_t to Y_t, but not from Y_t to X_t, it is consistent with the results in the time domain. This example clearly demonstrate that the complex Granger causality could detect the interactions between complexes.

Example 2 Consider the following model

$$\begin{cases} X_1(t) &=& 0.8X_1(t-1) - 0.5X_1(t-2) + aX_4(t-1) + \epsilon_1(t) \\ X_2(t) &=& 0.6X_2(t-1) - 0.4X_2(t-2) + \epsilon_2(t) \\ X_3(t) &=& 0.9025X_3(t-1) - 0.7X_3(t-2) + \epsilon_3(t) \\ X_4(t) &=& 0.3X_2(t) - 0.25X_3(t) + \epsilon_4(t); \end{cases}$$

where a is a constant.

In this example, we demonstrate that the interactions between nodes are too weak to be detected. But a combination of individual nodes can increase the interaction strength and thus can give arise to the complex interactions. In Fig. 2 **D** the data and their interactions are plotted when $a = .3$ with exact values are depicted in Fig. 2 **E**. To understand the reason, in Fig. 2 **F**, we plot the low bound of the confidence interval of the Granger causality as a function of a. In Example 2, we see that all the coefficient in $X_1(t)$ is of the order 0.1. When $a = 0.3$, the coefficient before $X_3(t-1)$ and $X_4(t-1)$ is 0.09 and 0.075, both below 0.1. Hence there is no interaction between X_3, X_4 and X_1. However, when a increases, the causality increases, and will soon become significant as shown in Fig. 2 **F** inset.

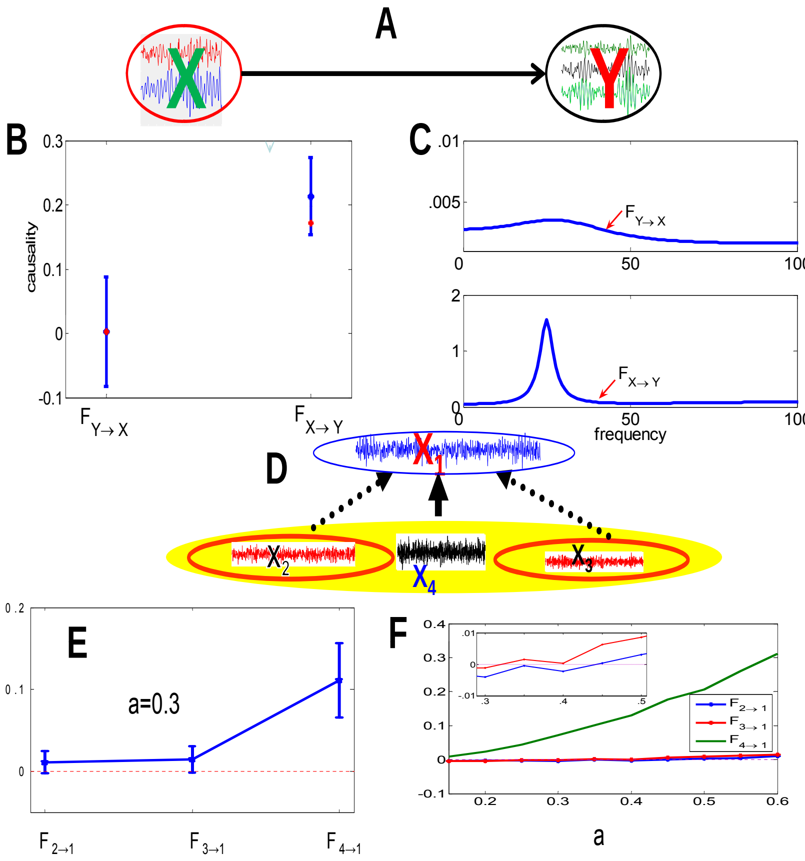

Figure 2: (**A**). Traces of the time series considered in Example 1 (inside ovals). The underlying causal relationship is represented by arrow. (**B**) Comparison between the time domain pairwise Granger causality and the frequency domain pairwise Granger causality. Blue line is the value and its confidence intervals of the pairwise complex Granger causality calculated in the time domain. Red dots are the summation (integration) of the pairwise complex Granger causality for frequencies in the range of $[-\pi, \pi]$. (**C**) is the results obtained in the frequency domain. The underlying causal relationships are shown in (**B**). (**D**) The pairwise complex Granger causality and their confidence intervals for Example 2. **E** The exact value of the Granger causality in Example 2 when $a = 0.3$. There are no causal relations between X_2 and X_1, and X_3 and X_1, but the causal relationship between X_4 and X_1 is significant. **F** The low bound of the causal relationship as a function of a. Inset clearly shows that when $0.25a \sim 0.1$ and $0.3a \sim 0.1$, the Granger causality between X_3 and X_1 and X_2 and X_1 becomes significant respectively.

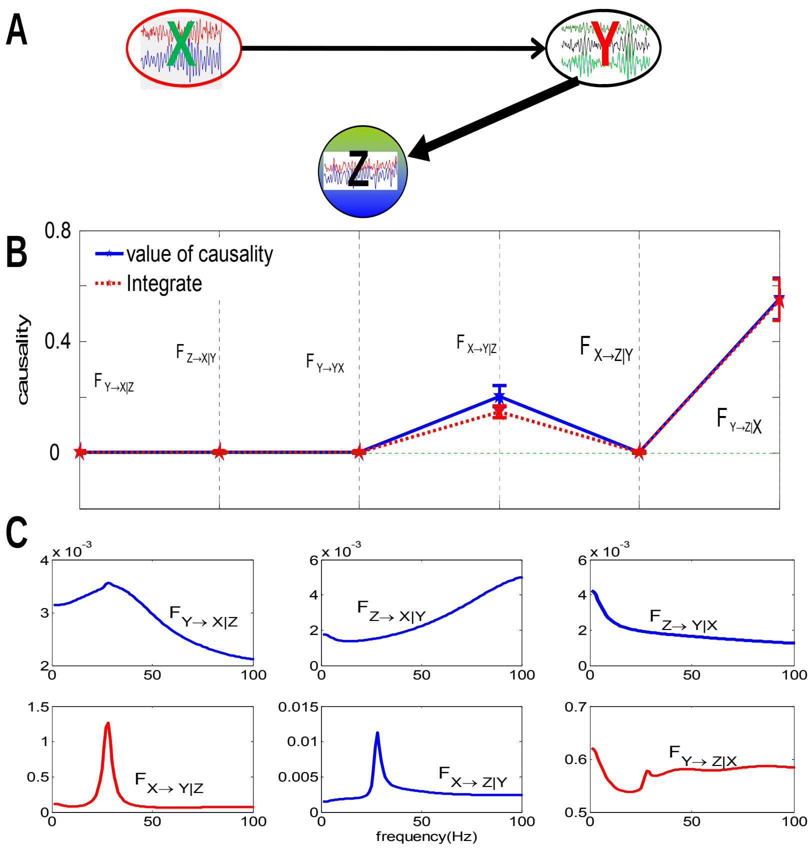

Figure 3: (**A**). A Traces of the time series considered in Example 3(upper). Y is shifted upward for visualization purpose. The underlying causal relationships are shown in (**B**). (**C**) The pairwise causality and their confidence interval. The replication times is 1000.

2.2 Synthesized data: partial complex interaction

Example 3 We modify the example 1 to the following model

$$\left\{\begin{array}{rcl} X_t &=& A_{11}X_{t-1} + A_{12}X_{t-2} + \overrightarrow{\epsilon_{1t}} \\ Y_t &=& A_{21}X_{t-1} + A_{22}X_{t-2} + B_{21}Y_{t-1} + \overrightarrow{\epsilon_{2t}} \\ Z_t &=& B_{31}Y_{t-1} + C_{31}Z_{t-1} + \overrightarrow{\epsilon_{3t}} \end{array}\right.$$

where $Z_t = (x_{6t}, x_{7t})^T$, $B_{31} = \begin{pmatrix} -0.95 & 0 & 0.1 \\ -0.5 & 0 & 0 \end{pmatrix}$, $C_{31} = \begin{pmatrix} 0.7 & 0.2 \\ 0.6 & 0 \end{pmatrix}$

Continuing from the example 1, we have another set of variables $Z_t = (x_{6t}, x_{7t})^T$.

Figure 3 A depicts the situation explained by the equations above. We can see the effect of partial complex interaction computed in the time domain in the Figure 3 B. The causal influence from $X \to Y|Z$ and $Y \to Z|X$ stand out as prominant connections between all other possibilities. Similarly in the frequency domain, the causalities computed are plotted in Figure 3 C. The plots again show the connections $X \to Y|Z$ and $Y \to Z|X$ to be more prominant from other connections.

2.3 A Circadian circuit

In Figure 4A, the top most gene ELF4 shows a strong circadian rhythm. It has the biggest M_{11} value. The importance of ELF4 in regulating the circadian activity is also reported in the literature [1, 6]. From the gene annotation (also presented in Supplemental material II), we found that ELF4 is related to two other genes: LHY and CCA1. ELF4 is necessary for light-induced expression of both CCA1 and LHY. Figure 4A plots the time trace of these genes. A circadian circuit related to LHY and CCA1 has been reported in the literature [30, 27]. The circuit comprises of three loops; PRR9, PRR7 and LHY/CCA1 in one loop (morning loop or loop III), TOC1 and GI as another loop (night loop or loop II), and a loop of LHY/CCA1, TOC1 and an unknown gene as loop I.

We therefore consider a gene circuit of 7 genes (see Supplemental material II). Four genes (ELF4, TOC1, LHY and CCA1) show a strong circadian rhythm as plotted in Figure 4B. We see that all genes have very strong magnitude on the period of 22 days. We applied partial Granger causality on those genes and the resulting network is shown in Figure 4C. The inferred structure is broadly in terms with the existing literature[7]. ELF4 plays an important role in regulating the circadian activity and is the most upstream genes. It interacts with both the loop III and the loop I. Loop III genes are closely interconnected via the interactions between PRR9, LHY and CCA1, and the interaction between CCA1 and PRR7. Similarly, in the loop I , TOC1 modulates LHY and CCA1. There are also links between loop III and loop I: PRR9 exerts influence on TOC1. TOC1 and PRR7 have a feedback loop. GI is an isolated gene in our structure, without having any interactions

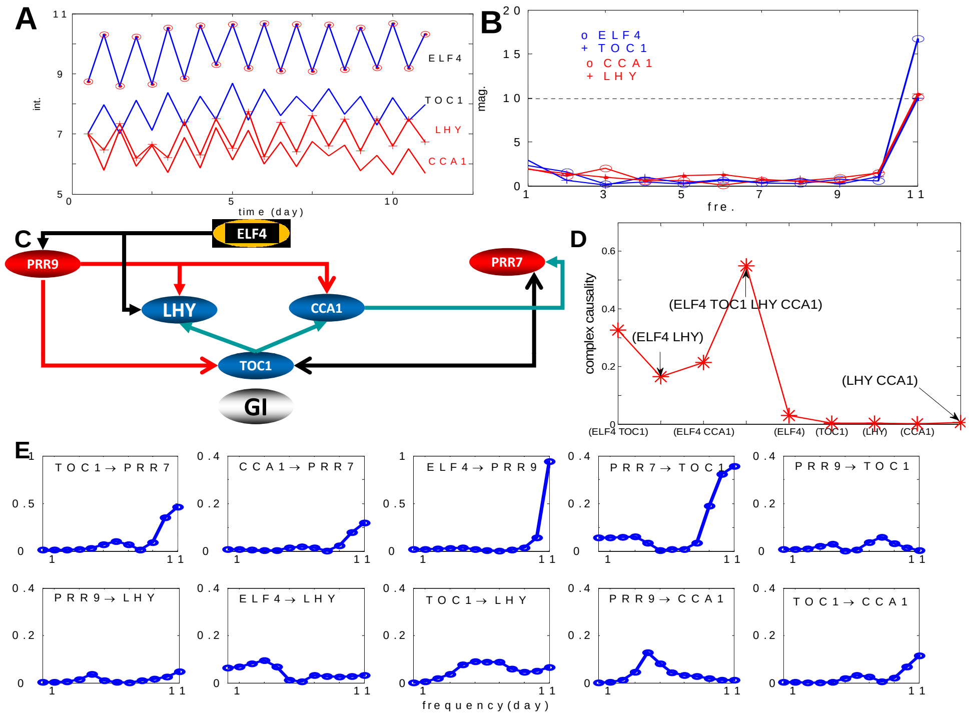

Figure 4: One gene circuit controlling circadian activity. A. Time trace of four genes, ELF4, TOC1, LFY and CCA1. ELF4 and TOC1 are in-phase oscillators, LFY and CCA1 are in-phase oscillators, but they are off-phase oscillators with respect to ELF4 and TOC1. B. Magnitudes vs. frequency for the four genes. They have highest magnitude at the frequency of one-day period. C. The gene circuit obtained in terms of PGC (see annotation in Supplemental material II). D. Complex interactions between different group of genes and GI. D. Gene interactions in the frequency domain.

with other six genes. In fact, this also coincides with the experimental findings. On page 4 [7], it is mentioned that *The gi single mutant had a relatively weak phenotype, whereas our assays of the triple gi; lhy;cca1 mutant demonstrate GI's importance*. This naturally leads us to introduce the notation of interactions between complexes as defined in Method section. Fig. 4**D** tells us that all single genes ELF4, TOC1, LHY, CCA1 and (LHY, CCA1) have very little influence on GI. However, ELF4, TOC1, LHY and CCA1 together exhibit a significant interaction with GI, which is in agreement with the experimental finding. We then analyse the interactions in the frequency domain. Not surprisingly, almost all the interactions show a 24 hour periodic behaviour by exhibiting a peak at one day period.

2.4 A metabolic network

Consider the following dynamic system:

$$\begin{cases} \dfrac{dS}{dt} &= -r_1 S + r_2 P_1 \\ \dfrac{dP_1}{dt} &= r_1 S - r_2 P_1 - r_3 P_1 + r_4 P_2 \\ \dfrac{dP_2}{dt} &= r_3 P_1 - r_4 P_2 \\ r_1 &= \dfrac{1}{1+\exp(-E)} \end{cases} \tag{45}$$

where E (enzyme) is the summation of a constant k and noise ω , $r_i, i = 1, 2, 3, 4$ are reaction rates, and r_2, r_3 and r_4 are constants. From the above equations, it is clear that the structure of such system is:.

$$S \rightleftharpoons P_1 \rightleftharpoons P_2 \tag{46}$$

with the reaction rate from S to P_1 is r_1, from P_1 to S is r_2, from P_1 to P_2 is r_3, and from P_2 to P_1 is r_4.

Figure 5 (upper plot) is the trace of three reactants S, P_1 and P_2, the reaction rate r_1 and enzyme E when the parameters are set to be $r_2 = 0.85, r_3 = 0.65, r_4 = 1.32k = 0.82, \omega$ is a Normal variable with variance 0.05^2. It is obvious that all these variables are stationary.

We can calculate the interaction from enzyme E to the reactants S, P_1 and P_2 and the reaction rate r_1. Figure 5 (bottom plot) plot the Partial Granger causality and its confidence interval after 1000 replications. It is easy to see that enzyme E has no influence to three

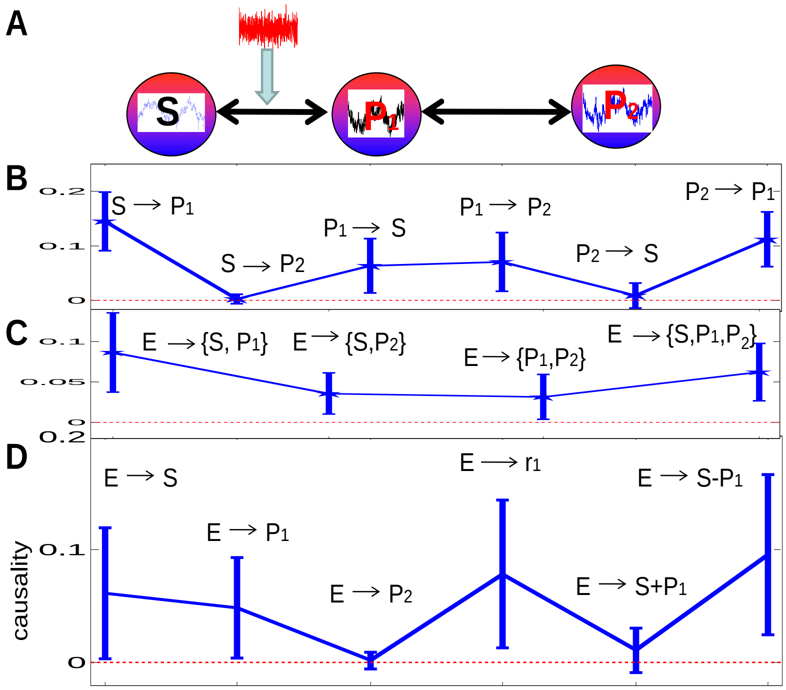

Figure 5: Upper plot: trace of three reactant S, P_1 and P_2, the reaction rate r_1 and enzyme E when the parameters are set to be $r_2 = 0.85, r_3 = 0.65, r_4 = 1.32, k = 0.82$, ω is a Normal variable with variance 0.05^2. The length of time points is 1000. Bottom plot: Partial Granger causality and its confidence interval after 1000 replications. Partial Granger causality and its confidence interval between three reactants S, P_1 and P_2 after 1000 replications.

reactant S, P_1 and P_2 individually, but it has influence on the reaction rate r_1 which is the case in real biology experiment. Since the reaction rate is unknown in real biological data, we then take a look at the complex Granger causality from enzyme E to reactants S and P_1. From the results, we found that E has influence on $S - P_1$ but has no influence on $S + P_1$. Also, we see from Figure 5 C that E influences S and P_1 grouped together. We can conclude from the above observations that E in the reaction between S and P_1 is acting on S. We also have the support to the claim made in the equations where r_1 and E are related, and r_1 is related to the reaction between S and P_1.

2.5 Impact of correlation on Granger causality

Actually, the complex interaction can, more significantly, also arise from the correlation between individual units. Let us consider a model where $y_i, i = 1, 2, \cdots, N$ are identical processes. The Granger causality from $(y_i(t), i = 1, \cdots, N)$ to $y(t)$ is $\log(1 + a2N(1 + (N-1)\rho))$ where ρ is the correlation coefficient between y_i. Fig. 6 illustrates how the complex interaction depends on the correlation. The individual unit has interactions (Fig. 6B, middle panel), but as a group, it could have (Fig. 6B, left panel) or have no interactions (Fig. 6B right panel). Not surprisingly, collaborative activity enhance the interaction (blue arrow in Fig. 6A), but antagonistic activity reduces or even diminishes the interaction.

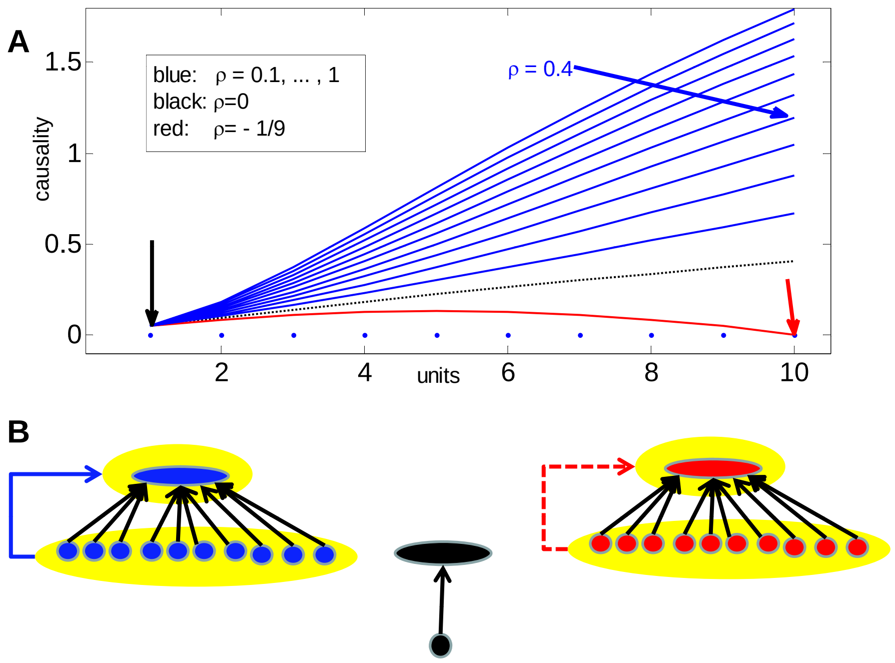

Figure 6: The role of correlation in the complex interaction. A. The Granger causality vs. units (N) for different with a=0.22. B. Three network structures (blue, black and red) correspond to the three arrows in A. Solid line indicates the existence of an interaction, but dashed line not.

3 Discussion

We considered a network of N units (genes, proteins, neurons etc. with intentions to reveal the interactions in the network. An interaction is defined as the causal relationship between two units. This is the driving force behind the current systems biology approach and the belief that the network interactions are the key for understanding many meaningful biology functions. For a network of N units, we may plausibly assume that there are N2 pairwise interactions (including self-interactions). Furthermore, a biological network is usually sparse and the total number of interactions could be smaller. Hence, with simultaneously recorded data at N units, we hope to be able to recover all interactions. Here we point out, however, that when using synthesized and biological datasets, the number of actual interactions should be of order exp(N) taking all possible subsets (complexes) of 1,2,. . .,N into account. This leads to an NP hard problem and a direct approach is bound to fail in revealing all the interactions.

References

[1] Doyle MR, Davis SJ, Bastow RM, McWatters HG, Kozma-Bogn LA, Nagy F, Millar AJ and Amasino RM (2002) The ELF4 gene controls circadian rhythms and flowering time in Arabidopsis thaliana *Nature* **419**, 74-77.

[2] Geweke J. (1982) Measurement of Linear Dependence and Feedback Between Multiple Time Series. *Journal of the American Statistical Association*, **77**: 304-313.

[3] Geweke J. (1984) Measures of Conditional Linear Dependence and Feedback Between Time Series. *Journal of the American Statistical Association*, **79**: 907-915.

[4] Granger C. (1969) Investigating Causal Relations by Econometric Models and Cross-spectral Methods. *Econometrica*, **37**: 424-438.

[5] Guo SX, Wu J, Ding MZ, Feng JF. (2008) Uncovering Interactions in the Frequency Domain.(supplementary material I) *PLoS Comput Biol.*,**4(5)**: e1000087.

[6] McWatters HG, Kolmos E, Hall A, Doyle MR, Amasino RM, Gyula P, Nagy F, Millar AJ, Davis SJ (2007) ELF4 Is Required for Oscillatory Properties of the Circadian Clock, *Plant Physiology* **144**: 391-401.

[7] Locke J. C. W., Kozma-Bognar L., Gould P. D.,Feher B., Kevei E., Nagy F., Turner M. S.,Hall A., Millar A. J. (2006), Experimental validation of a predicted feedback loop in the multi-oscillator clock of Arabidopsis thaliana, *Molecular Systems Biology*, 2, 59.

[8] Wiener.N. (1956) *The theory of prediction*. In: Beckenbach EF (ed) Modern mathematics for engineers, chap 8. McGraw-Hill, New York.
